# Mitochondria in endothelial cells angiogenesis and function: current understanding and future perspectives

**DOI:** 10.1186/s12967-023-04286-1

**Published:** 2023-07-05

**Authors:** Zhen Luo, Jianbo Yao, Zhe Wang, Jianxiong Xu

**Affiliations:** 1 Shanghai Key Laboratory of Veterinary Biotechnology/Shanghai Collaborative Innovation Center of Agri-Seeds, School of Agriculture and Biology, Shanghai Jiao Tong University, Dongchuan Road 800, Minhang District, Shanghai, China; 2 Division of Animal and Nutritional Sciences, West Virginia University, Morgantown, West Virginia USA

**Keywords:** Mitochondrial protein, Angiogenesis, Endothelial cells, Signaling pathways, Cardiovascular diseases

## Abstract

Endothelial cells (ECs) angiogenesis is the process of sprouting new vessels from the existing ones, playing critical roles in physiological and pathological processes such as wound healing, placentation, ischemia/reperfusion, cardiovascular diseases and cancer metastasis. Although mitochondria are not the major sites of energy source in ECs, they function as important biosynthetic and signaling hubs to regulate ECs metabolism and adaptations to local environment, thus affecting ECs migration, proliferation and angiogenic process. The understanding of the importance and potential mechanisms of mitochondria in regulating ECs metabolism, function and the process of angiogenesis has developed in the past decades. Thus, in this review, we discuss the current understanding of mitochondrial proteins and signaling molecules in ECs metabolism, function and angiogeneic signaling, to provide new and therapeutic targets for treatment of diverse cardiovascular and angiogenesis-dependent diseases.

## Introduction

Angiogenesis (sprouting) is a multi-stage process involving creation of new vessels from existing ones, increase of vascular permeability, degradation of surrounding basement membrane by release of proteolytic enzymes, followed by proliferation and migration of endothelial cells (ECs), tube-like structure formation, and recruitment of mural cells, such as pericytes, to maintain vessel maturation, stabilization and blood flow. It plays essential roles in wound healing, female reproductive system, embryonic development and placentation [1, 2]. ECs are indispensable for vascular homeostasis, remodeling and angiogenesis, while ECs dysfunction is a hallmark and pathogenesis of many cardiovascular diseases, such as hypertension and atherosclerosis [3]. Thus, understanding the potential molecular mechanisms regulating ECs metabolism, function and angiogenesis provides preventive and therapeutic strategy for multiple physiological processes and vascular diseases. Given the growing evidence regarding mitochondrial biology and signaling in ECs angiogenesis, this review summarizes and discusses the mitochondria-related proteins and signaling molecules that participate in ECs metabolism, function and angiogenesis.

## Mitochondria in ECs angiogenesis

Many different types of cells reside in the vascular system including ECs, smooth muscle cells, fibroblasts, pericytes and vascular stem cells. ECs line the inner vessel walls that safeguard transport logistics, and regulate vascular permeability and tone. ECs can be divided into tip cells, stalk cells and quiescent phalanx cells according to their functions during blood vessel formation [4, 5]. Tip cells are responsible for migration including sprout initiation, elongation and anastomosis. Stalk cells synthesize cellular components for proliferation and growth, while phalanx cells are quiescent and non-proliferating [6]. Angiogenic factors such as VEGF binding to multiple receptors (VEGFR-1, -2, and -3) in the tip cells, induces receptors dimerization and autophosphorylation and activates downstream pathways such as MAPK and PI3K-Akt, which is responsible for ECs proliferation, migration and tube formation. Also, the receptors mediated signals promotes the expression of the Delta-like ligand (DLL)4/NOTCH pathways in stalk cells, which provide a feedback loop to limit excessive angiogenesis in response to VEGF [7].

Mitochondria are the intracellular central sites responsible for oxygen consumption and ATP production that play a pivotal role in cell signaling and metabolic regulation. It is reported that mitochondria contribute to 15% ATP production in ECs, while most of the energy supplies rely on aerobic glycolytic pathways [8]. ECs resemble cancer cell, which are low oxygen consumption and highly adaptive to proliferate, migrate and survive under hypoxia compared with other cell types [9]. Despite low oxygen consumption, mitochondria have been considered as sensors and integrators of environmental stress in ECs [10]. Furthermore, increasing evidence has suggested the importance of mitochondrial biology including metabolism, quality control, location, signaling regulation and homeostasis in controlling ECs permeability, tone, migration and proliferation under physiological and pathophysiological conditions [6, 11, 12]. Here, the major mitochondria-related proteins and signaling molecules in regulating ECs metabolism, function and angiogenic signaling homeostasis were summarized (Table 1).

**Table 1:** Mitochondrial-related proteins that involved in angiogenesis and endothelial function in various ECs, animal models or clinical trials under different stimulations were reviewed and summarized

Localization	Protein	Stimuli	Role in angiogenesis	Cell types	Animal model/clinical trials	References
^OMM^						
	SIRT3	CRIF1 deficiency, high glucose, hypoxia, angiotensin II	SIRT3 knockout exhibited higher ROS formation and OCR, decreased PFKFB3−dependent glycolysis, reduced VEGF and angiogenesis; suppressed reendothelialization capacity in EPCs, induced premature senescence, accelerated Ang II−induced EndoMT. SIRT3 overexpression promotes proliferation, migration, vessel sprouting and tube formation through VEGFR3 and ERK pathways and PINK1/Parkin−mediated mitophagy	MECs, EPCs CMECs, LECs, HUVECs, MAECs	Hypertension patients, KO and OE mice	[204, 205, 207–210]
	SIRT4	LPS	SIRT4 knockdown increased the pro−inflammatory cytokines IL−1β, IL−6 and IL−8 through activation of NF−kB, promoted MMP−9 and ICAM−1, while overexpression reversed these factors	HUVECs		[216]
	SIRT5	Ischemia–reperfusion, hypoxia	Downregulation of SIRT5 induced mitochondrial dysfunction, decreased angiogenic capacity and endothelial permeability, upregulated occludin and claudin−5, induced capillary rarefaction	HBMECs EPCs	KO mice, hypertensive patients	[221–223]
	Akap1	Hypoxia, femoral artery ligation	Akap1 inhibited VEGFR2 degradation through PKA/p38−dependent p−VEGFR2 at Y1173, Akap1 knockout decreased cell migration and proliferation, impaired blood flow and capillary density	HUVECs	KO mice	[242, 243]
	VDAC1		VDAC1 knockdown decreased ATP production and increased the AMP:ATP ratio, which in turn activated AMPK and phosphorylated raptor, inhibited mTOR activity and cell proliferation	HUVECs		[92]
	TSPO	Laser, hypoxia	TSPO KO decreased retina pro−angiogenesis and vascular leakage, increased glioma growth and angiogenesis by promoting glycolysis and reducing oxidative phosphorylation,	Phagocyte, GL261 cells, GBM1B cells	TSPO^KO^ mice	[116, 117]
	Drp1	Caffeine, hypoxia, replicative senescence, ischemia–reperfusion	Drp1 knockdown decreased lamellipodia formation, cell migration and proliferation via mitochondrial Ca^2+^ dependent pathway and impairment of autophagic flux. Drp1−C644A improved wound healing and angiogenesis in PDIA1 deficient mice. Inhibition of Drp1 phosphorylation at Ser616 preserving ischemia-reperfusion injury	HUVECs, PAECs CMECs	Mice under hindlimb ischemia, PDIA1 deficient mice, BI1 transgenic mouse	[167–171]
	Mfn1/2	VEGF	Knockdown of Mfn2 affected mtROS production, while knockdown of Mfn1 reduced NO signaling. Mfns are not regulated by angiogenic cues and dispensable for developmental angiogenesis	HUVECs, MPECs	Mfn1^KO^ mice	[164, 165]
	Fis1	Replicative senescence	Downregulation of Fis1 induced senescence and mitochondrial dysfunction, and impaired EPCs activity	EPCs	Hindlimb ischemic mice	[172]
	PINK1		PINK1 knockout reduced cardiac capillary density, increased oxidative stress and impaired mitochondrial function	Cardiomyocyte	KO mice	[144]
	Parkin		Parkin overexpression decreased eNOS expression and induced mitochondrial dysfunction by ubiquitination of ERRα	MAECs		[147]
	FUNDC1	VEGF	Deletion of FUNDC1 disrupted MAM formation and angiogenesis dependent on VEGFR2 expression through decreasing the binding of SRF to VEGFR2	HUVECs	EC−specific FUNDC1^KO^ mice	[154]
	BNIP3	Hypoxia	BNIP3 showed antagonistic effect with VEGF on ECs apoptosis under hypoxia	HPAECs, HUVECs and HLMECs		[163]
IMM						
	^p66shc^	^Streptozotocin, VEGF, high glucose, age, ox−LDL, LDLC^	p66shc KO mice showed upregulation of eNOS and HO−1, prevented streptozotocin−induced endothelial dysfunction and oxidative stress. VEGF stimulation promotes pS36−pp66Shc formation through ERK/JNK/PKC, which involved in VEGF−induced VEGFR2 autophosphorylation. P66shc knockdown inhibited glucose−induced Rac1 activation and mitochondrial damage. P66shc inhibited the Ras−PI3K−Akt−eNOS−NO production. Acetylation of p66Shc promoted its p-S36. LDLC increased CpG hypomethylation and acetylation of histone 3 of p66shc promoter	HUVECs, HRECs, HAECs	p66shc^KO^ mice,	[36, 37, 39, 40, 42, 44, 45]
	UQCRB	Antimycin A	Inhibition of UQCRB reduced complex III enzyme activity, blocked mtROS−mediated VEGFR2, reduced EC proliferation, OCR and NAD^+^/NADH ratio, but not migration	HUVECs, QPC^KO^ lung ECs	QPC^KO^ mice	[49, 50]
	PHB1	VEGF, TGF−β1	Knockdown of PHB1 resulted in mitochondrial dysfunction and ROS production via inhibition of complex I, led to cytoskeletal rearrangements and cell senescence by increasing Akt and Rac1, reduced cell migration and tube formation. Activation of PHB ameliorated TGF−β−induced EndoMT	BAECs, HUVECs, HMECs	Transverse aortic constriction	[180, 181]
	UCP2	VEGF, ischemia, hypoxia	Overexpression UCP2 promoted tube formation in MAECs and BAECs, while knockdown UCP2 increased VEGFR2 phosphorylation and cell proliferation in HRMECs	MAECs, BAECs, HRMECs, MLECs	OIR model rat, AMPKα^KO^ mice, UCP OE and KO mice, MnSOD^+/−^ mice	[186, 188, 189]
	FECH	Hypoxia	FECH depletion decreased proliferation, migration and tube formation, suppressed p−VEGFR2 and VEGFR2, eNOS and HIF−1α, but did not affect macrovascular HUVECs proliferation	HRECs, HUVECs, HBMECs	L−CNV mice, Fechm1Pas mice model, OIR model	[247–249]
	OPA1		Promotion of angiogenesis by inhibiting NF−kB and maintaining cytosolic Ca^2+^ homeostasis through MCU1	HUVECs, MPECs	Opa1^iΔEC^ mice, OPA1^TG^ mice, Opa1^ΔEC/ΔEC^ mice	[164]
^Matrix protein^						
	MnSOD	High glocuse, non−reperfused myocardial infarction	H_2_O_2_ production by MnSOD promoted VEGF expression, cell sprouting and blood vessel formation by oxidation of PTEN. Knockdown of MnSOD reduced diabetic wound healing assays, MnSOD gene therapy restored angiogenesis and wound repair in diabetic mice. MnSOD promoted ECs proliferation and coronary angiogenesis, protected cardiac function in non−reperfused myocardial infarction	BLMC, EPCs MHECs	Diabetic mice, OE mice	[14–16]
	IDH2		IDH2 knockdown decreased expression of mitochondrial function, eNOS/NO production, induced endothelial inflammation via p66shc−mediated mitochondrial oxidative stress	HUVECs, MS1 cells, MLECs	IDH2^KO^ mice	[74, 75]
	ALDH2	Acetaldehyde, ischemia, hypoxia, β−amyloid, ethanol	Promotion of migration, proliferation and angiogenesis through improving mitochondrial function and HIF−1α−/VEGF−dependent mechanism. Hyperacetylation promoted ethanol−induced Akt−eNOS activation	HUVECs, HAECs	ALDH2^KO^ mice, CTO patients	[54, 57–59]
	CypD	VEGF, angiotensin II, IL17A, TNFα	CypD−deficient increased VEGF−induced proliferation and angiogenesis, while S−glutathionylation of CypD increased ROS production	HPAECs HPMECs, HAECs	CypD^KO^ mice	[100, 101]
Unknown						
	FAM3A	Ischaemia, hypoxia, CoCl_2_	Promoted capillary density and angiogenesis by enhancing CREB−dependent VEGFA transcription through ATP/P2 receptor/Ca2+ pathway	HUVECs	Hind limb ischaemia mice	[120]
	Trx2	VEGF, hypercholesterolemia, ischemia, TNF	Promotion of cell migration and survival by increasing NO bioavailability and inhibiting oxidative stress and ASK1−induced apoptosis	MAECs, MLMECs BAECs, HUVECs	Trx2^TG^ mice, ApoE−deficient mice, eNOS^KO^, and eNOS^KO^/Trx2^TG^ mice	[65–68]
	Cyp1B1		Cyp1B1 deficient impaired revascularization, eNOS and migration, increased oxidative stress and thrombospondin−2 in RECs, but increased VEGFR2 expression, cell proliferation and migration in LSECs	RECs, LSECs	Cyp1B1^KO^ mice	[229, 235]
	NRP1	Iron	NRP1 prevented iron−dependent mitochondrial superoxide production and premature senescence through interacting with the ABCB8	HMECs	NRP1^ECKO^	[264]

MECs: microvascular endothelial cells; LECs: lung endothelial cells; HBMECs: human brain microvascular ECs; RMECs: retinal microvascular endothelial cells; MLECs: murine lung endothelial cells; HUVECs: human umbilical cord vein endothelial cells; PAECs: pulmonary artery endothelial cells, EPCs: endothelial progenitor cells; MAECs: mouse aortic endothelial cells; HPAECs: human pulmonary artery endothelial cells; HLMEC: human lung microvascular endothelial cells; BAECs: bovine aortic endothelial cells; LMECs: lung microvascular endothelial cells; CMECs: cardiac microvascular endothelial cells; MLMECs: mouse lung microvessel endothelial cells; HRECs: human retinal microvascular endothelial cells; HPMECs: human pulmonary microvascular endothelial cells; REC: retinal endothelial cells; LSEC: liver sinusoidal endothelial cells; MHECs: mouse heart endothelial cells; BLMCs: bovine lung microvessel cells; HAECs: human aortic endothelial cells; HMECs: human microvascular endothelial cells; MPECs: mouse pulmonary endothelial cells; MS1: mouse islet endothelial cells; PDIA1: protein disulfide isomerase A1; BI1: bax inhibitor 1; QPC: a subunit of the respiratory chain complex III. OIR: oxygen-induced retinopathy; L-CNV: laser-induced choroidal neovascularization; Fech^m1Pas^: a partial loss-of-function M98K point mutation in the Fech gene; OPA1^iΔEC^: an inducible endothelial knockout OPA1 mice. OPA1^ΔEC/ΔEC^: EC Opa1 knockout mice; CTO patients: patients with chronic total occlusion. ABCB8: ATP-binding cassette B8. LDLC: low-density lipoprotein cholesterol; oxLDL: oxidized low density lipoprotein; MAM: mitochondria-associated endoplasmic reticulum membranes. SRF: serum response factor

## Mitochondrial ROS (mtROS) in ECs angiogenesis

**Fig. 1:**
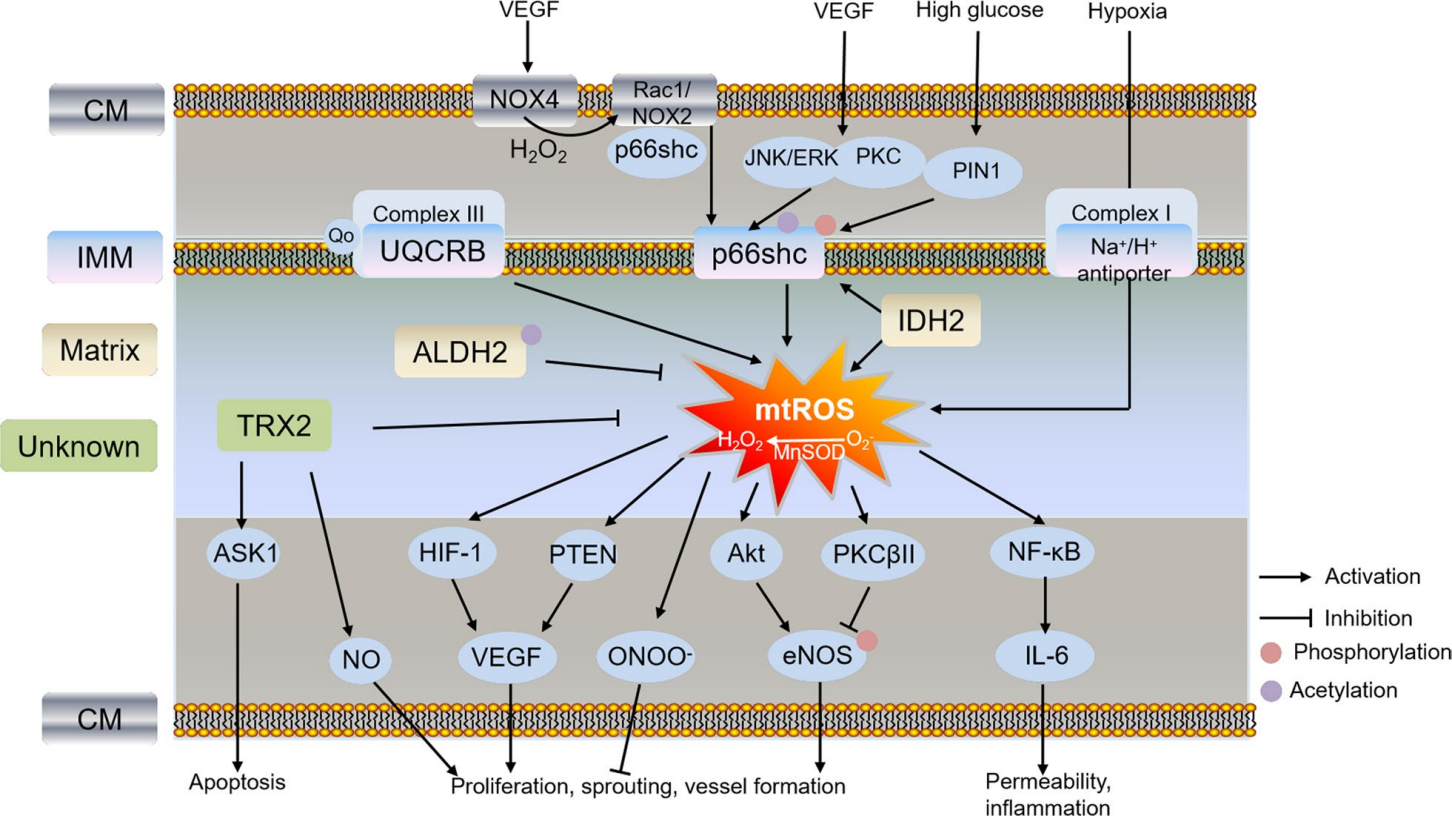
Schematic pathway of mitochondrial ROS and related protein in regulating ECs function and angiogenesis under stress conditions. Different colors of boxes indicate different locations of protein in cells listed on the left. VEGF induced ECs migration and angiogenesis through increasing phosphorylated p66Shc (Ser36) by activation of NOX4-H_2_O_2_-NOX2-mtROS, or JNK/ERK and PKC pathways. Phosphorylated p66Shc also increased its localization to the mitochondria through interacting with PIN1 under high glucose treatment. The mechanism of p66shc-mediated endothelial dysfunction including HIF-1α/VEGF, PTEN/VEGF, ONOO-, Akt/PKCβ-eNOS-mediated ECs proliferation, sprouting and vessel formation, or NF-κB-mediated ECs permeability and inflammation regulated by mtROS; Trx2 increased cell migration and survival by increasing NO bioavailability and inhibiting ASK1-induced apoptosis. Mitochondrial proteins such as UQCRB, ALDH2, IDH2 and Na^+^/H^+^ antiporter involved in EC angiogenesis and function through mtROS-mediated pathways. p66shc: 66 kDa proto-oncogene Src homologous-collagen homologue adaptor protein; UQCRB: ubiquinol-cytochrome c reductase binding protein; TRX2: thioredoxin 2; ASK1: apoptosis signaling kinase-1; ALDH2: aldehyde dehydrogenase 2; IDH2: isocitrate dehydrogenase 2; PIN1: prolyl isomerase peptidyl-prolyl cis–trans isomerase NIMA-interacting 1; Rac1: Rho-related small GTPase 1; eNOS: endothelial nitric oxide synthase; PTEN: phosphatase and tensin homolog deleted from chromosome 10; CM: cell membrane; OMM: outer mitochondrial membrane; IMM: inner mitochondrial membrane

It is commonly accepted that the mediators of ROS production and redox signaling regulate ECs function, angiogenesis and remodeling through several signaling pathways [13]. MtROS was reported to have diverse actions on endothelial function (Fig. 1). Mitochondrial generation of H_2_O_2_ by MnSOD promoted VEGF expression, cell sprouting and blood vessel formation in vitro and in vivo by oxidation of phosphatase and tensin homolog deleted from chromosome 10 (PTEN), which was attenuated by catalase (CAT) coexpression, indicating for the first time that mitochondrial H_2_O_2_ was positively involved in angiogenic switch [14]. Indeed, previous investigations demonstrated that optimization of mitochondrial matrix protein MnSOD expression may promote mature capillary formation and angiogenesis. Endothelial progenitor cells (EPCs) are precursor of vascular ECs, which can be transported to injury sites, forming new blood vessels and promoting wound healing. MnSOD gene therapy could improve EPCs-mediated angiogenesis and wound healing [15]. Recently, by using a novel transgenic animal model that overexpression of MnSOD in mouse heart ECs (MHECs), MnSOD promoted ECs proliferation and coronary angiogenesis, protected cardiac function in non-reperfused myocardial infarction by reduction of mtROS, increase of assembly of mitochondrial complexes into supercomplexes, and upregulation of mitochondrial respiration [16]. Furthermore, natural compounds such as resveratrol [17], and barley beta-glucan [18] could also be sufficient to induce endothelial MnSOD-mediated angiogenesis, indicating MnSOD-dependent therapy are effective to promote ECs angiogenesis and prevent vascular diseases.

Extracellular VEGF could increase ECs permeability, stimulate proliferation and promote migration through increase of mtROS, activation of Rho-related small GTPase 1 (Rac1) and downstream pathways such as PAK, Akt, p38 and ERK, indicating that mtROS plays a critical role in VEGF-dependent angiogenesis [19]. Indeed, NADPH oxidases (NOX) has long been considered as the major source of ROS responsible for angiogenesis in ECs [20]. mtROS was also involved in NOX (NOX2 and NOX4)-dependent angiogenesis in ECs, which has been reviewed previously [21]. NOX4-derived H_2_O_2_ partly activated NOX2 to promote mtROS production via phosphorylation of p66Shc at Ser36, thereby promoting VEGF-induced ECs migration and angiogenesis. It represents a novel feed-forward mechanism of angiogenic signaling, which also called “ROS-induced ROS release” [22, 23]. By using Tet-NOX2 conditional transgenic mice, Shafique et al., demonstrated that NOX2-derived ROS resulted in increased mtROS in MHECs. Furthermore, short-term increase of mtROS promoted proliferation and angiogenic sprouting through activation of endothelial nitric oxide synthase (eNOS) in ECs, while long-term increase of mtROS resulted in ONOO^−^ formation and inactivation of MnSOD by nitrotyrosine, decreased membrane potential, and inhibited ECs proliferation, indicating mtROS plays dual roles in the NOX-dependent endothelium function and angiogenesis [24].

Another important regulator of mtROS mediating ECs function and angiogenesis is hypoxia-inducible factors (HIFs). Under normoxia, following hydroxylation of prolyl hydroxylase 2 (PHD2) and polyubiquitination by the von Hippel-Lindau (VHL) ubiquitin ligase, HIF-1α is targeted for degradation by the proteasome. While hypoxia inactivates PHD2, which promotes the stabilization of HIF-1α and its translocation to the nucleus, resulting in its binding to hypoxia-responsive elements (HRE) in the promoter region of target genes and transcriptionally activating their expression [25]. Importantly, iron, 2-oxoglutarate and ROS levels might regulate the activity of PHDs and hydroxylation of HIF-1α protein. MtROS production from the Qo site of mitochondrial complex III is required for HIF-1α stabilization, while scavenging superoxide from complex III by S3QELs decreased hypoxia-induced stabilization of HIF-1α without affecting oxidative phosphorylation [26, 27]. Indeed, inhibition of mtROS generation is a common mechanism of HIF-1 inhibitors [28]. All these results suggest that mtROS from complex III senses hypoxia signaling and regulates HIF-1 expression.

ECs are highly adaptive to hypoxia [9]. Hypoxia was reported to increase mtROS production, but not increased ROS production from NOX, xanthine oxidase or NO synthase. The increased mtROS activated NF-κB and secreted IL-6 in HUVECs, resulting in increasing endothelial permeability [29]. Acute hypoxia also transiently induced complex I deactivation and superoxide production through its Na^+^/H^+^ antiporter activity in bovine aortic ECs (BAECs) [30, 31], indicating complex I is also the site responsible for mtROS production in ECs under hypoxia. Furthermore, a new regulatory mechanism of mtROS production in cell adaptation to hypoxia has been reported. Na^+^/Ca^2+^ exchanger (NCLX)-mediated import of Na^+^ interacted with phospholipids, reduced fluidity of inner mitochondrial membrane (IMM) and mobility of free ubiquinone between complex II and complex III, consequently promoting superoxide formation, regulating hypoxia signaling and mitochondrial function [32]. Although ROS-HIF-1α is an important pathway to regulate ECs permeability and angiogenesis, the exact mechanism that mtROS activates HIF-1α is not fully understood. Oxidation modification of cysteine residues within PHD2, and regulation of ferric iron as cofactors by PHD and factor inhibiting HIF (FIH) are potential mechanisms responsible for HIF-1α protein stabilization [33, 34]. Thus, understanding the interactions and specific mechanism between mtROS and HIF-1 will promote the development of more specific therapeutic agents for improving ECs function and treatment of vascular diseases.

### p66Shc

p66Shc, a 66 kDa proto-oncogene Src homologous-collagen (Shc) homologue adaptor protein, is one of the three family members of Shc along with p46Shc and p52Shc. Unlike p52Shc and p46Shc, p66Shc showed varied expression among tissues. p66Shc is ubiquitously expressed in cytosol, endoplasmic reticulum and inner-mitochondrial space that regulation of oxidative stress signals and apoptosis pathways. Mice lacking p66shc displayed prolonged lifespan, increased resistance to oxidative stress and vascular apoptosis, increased antioxidant enzyme heme oxygenase 1 and eNOS, preventing atherogenesis and diabetes-induced endothelial dysfunction and vascular diseases [35, 36]. p66Shc played a critical role in ROS-dependent VEGF signaling and angiogenesis in ECs. VEGF not only promoted p66Shc phosphorylation at Ser36 by activation of JNK/ERK or PKC pathway, but also activated NOX2 component Rac1 by increasing its binding to nonphosphorylated p66Shc, resulting in ROS-dependent VEGF receptor 2 (VEGFR2) phosphorylation, which stimulated ECs migration and proliferation [37]. Furthermore, Ser36 is a critical regulatory site of p66Shc for its localization to mitochondria, resulting in increased mtROS generation and apoptosis. p66Shc also displayed a link between mitochondrial and cytosolic ROS during development of diabetic retinopathy. High glucose increased cytosolic p66Shc expression and its binding with Grb2 in human retinal ECs (HRECs), which released Sos1 from Sos1-Grb2 complex, activated Rac1/NOX2 by altering the GEF binding of Sos1, and increased cytosolic ROS production. In addition, phosphorylated p66Shc increased its localization to the mitochondria through interacting with prolyl isomerase peptidyl-prolyl cis–trans isomerase NIMA-interacting 1 (PIN1), and increased mtROS production [38].

Another important pathway of p66shc-mediated endothelial dysfunction is eNOS/NO. Overexpression of p66shc inhibited Ras-PI3K-Akt-eNOS/NO pathways, while knockdown of p66shc increased eNOS phosphorylation at S1177 and NO production, decreased O_2_^−^ production, protecting against endothelial dysfunction induced by age and oxidized low-density lipoprotein (ox-LDL) [39, 40]. Furthermore, p66Shc-derived ROS production inhibited phosphorylation of eNOS at T495 through activation of PKCβII, indicating different regulatory site of eNOS mediated by p66shc in ECs [41]. Interestingly, eNOS uncoupling played a crucial role in p66Shc-mediated ROS generation. Inhibition of eNOS in primary human aortic ECs (HAECs) increased p66Shc Ser36 phosphorylation under basal conditions, while inhibition of eNOS reduced p66Shc Ser36 phosphorylation under ox-LDL treatment [42]. These studies suggested different regulatory roles of eNOS mediated by p66shc were affected by different stress conditions. In addition, transcriptional and post-translational modification of p66shc also participated in endothelial function. Transcriptional downregulation of p66shc by inhibition of p53 alleviated angiotensin II-induced impairment of endothelium vasorelaxation [43]. Acetylation of p66Shc at Lys81 by SIRT1 under high glucose condition promoted its phosphorylation on Ser36 and translocation to mitochondria, and increased mtROS production, suggesting regulation of p66Shc Lys81 acetylation is important target for inhibiting mtROS production and improving endothelial dysfunction [44]. In addition, high glucose and LDL increased p66Shc expression by promoting its promoter CpG hypomethylation and histone 3 acetylation in HAECs and HUVECs [41, 45]. These results indicated that targeting p66shc is a critical way to regulate mtROS production, angiogenesis and ECs function through multiple mechanisms. Although Cys59-mediated thiol-disulfide interaction and copper-dependent cyt c oxidation are responsible for p66shc-mediated ROS production, the specific mechanism is not fully characterized [46]. Thus, exploring the mechanism of p66Shc-mediated ROS production and metabolism in ECs will provide new strategies to prevent and treat endothelial dysfunction and vascular diseases.

### Ubiquinol-cytochrome c reductase binding protein (UQCRB)

UQCRB is a conserved subunit of the mitochondrial complex III proteins that regulate mitochondrial electron transport and cellular oxygen sensing by modulating ROS production [47]. UQCRB was reported to be involved in hypoxia-induced ROS generation, HIF and VEGF activation, and angiogenesis in tumor and zebrafish [47, 48]. In HUVECs, inhibition of UQCRB by siRNA or inhibitor terpestacin reduced VEGF-mediated cell proliferation, invasion and tube-formation through mtROS production [49]. Furthermore, depletion of UQCRB impaired ECs proliferation by decreasing NAD^+^/NADH but not its migration, diminished amino acid levels but did not affect genes involved in anabolism or nucleotide levels [50]. These results indicated that mitochondrial complex III serves as biosynthetic site for ECs proliferation. In addition, a few natural small molecules specifically targeting UQCRB have been identified including terpestacin [47], oxymatrine [51] and 6-(1-Hydroxynaphthalen-4-ylamino)dioxysulfone)-2H-naphtho[1,8-bc]thiophen-2-one (HDNT) [52], indicating targeting mitochondrial complex III through UQCRB is a new strategy for treatment of tumor and angiogenesis-related diseases.

### Aldehyde dehydrogenase 2 (ALDH2)

ALDHs are a superfamily that includes 19 subtypes in humans. Mitochondrial matrix protein ALDH2 acts as an indirect antioxidant that detoxifies aldehydes such as malondialdehyde (MDA) and 4-hydroxynonenal (4-HNE), which reduces their cytotoxicity and prevents from oxidative injury. ALDH2 polymorphism or mutation is associated with increased risk of cardiovascular disease, diabetic complications and neurodegenerative diseases, indicating it is a promising therapeutic target in treatment of many diseases [53–56]. ALDH2 deficiency induced cardiovascular oxidative stress, while overexpression of ALDH2 prevented acetaldehyde-, β-amyloid-induced oxidative injury and promoted ECs migration, proliferation and angiogenesis through improving mitochondrial function and HIF-1α/VEGF-dependent mechanism [54, 57, 58]. Hyperacetylation of ALDH2 by poly(ADP-ribose) polymerase (PARP)-SIRT3 inactivation promoted ethanol-induced Akt-eNOS activation, and ROS-induced HAECs injury [59], indicating acetylation of ALDH2 also plays an important role in ECs function. In addition, cell therapy based on ALDH2 activity represents a promising strategy for improving angiogenesis, but different outcomes have been obtained. Bone marrow cell transplantation with high ALDH activity improved perfusion, capillary density and revascularization in ischemic limbs but had no significant effect in ALDH2-knockout mice [54, 60]. ALDH2 activity is present at high levels and also represents a reliable indicator of vascular ECs precursor EPCs [61]. These results suggested ALDH2 may be a potential pro-angiogenic target for treatment of cardiovascular diseases, but the application of therapeutic strategy still has a long way to go. Of note, overexpression of ALDH2 prevented hypoxia-induced pulmonary hypertension by lowering phosphorylation of Drp1 at Ser616 and cell proliferation in smooth muscle cells but not in ECs [62]. Since both pulmonary arterial smooth muscle cells and arterial ECs play pivotal role in vascular remodeling, the differential regulatory mechanisms of ALDH2 in these cell types under diseases need further study.

### Thioredoxin 2 (Trx2)

Thioredoxin systems including both thioredoxin 1 (Trx1) and thioredoxin (Trx2), localized in cytosol and mitochondria, are evolutionarily conserved antioxidant and molecular chaperone [63]. Both Trx1 and Trx2 are responsible for protecting against oxidative stress, apoptosis, and maintaining the cellular metabolism, which are reduced by thioredoxin reductases (TrxR) and inhibited by thioredoxin-interacting protein (TXNIP). Indeed, the thioredoxin systems including Trx, TrxR and TXNIP are involved in ECs proliferation, migration and angiogenesis, which has been reviewed previously [63]. Furthermore, Trx1 was reported to promote HIF-1α synthesis while Trx2 decreased HIF-1α translation, suggesting their opposite roles on HIF-1α expression [64]. Trx could bind apoptosis signaling kinase-1 (ASK1) and induce ASK1 ubiquitination/degradation that is not dependent on its redox activity sites (C32 and C35) in ECs, while overexpression of Trx2 increased cell migration and survival by increasing NO bioavailability and inhibiting ASK1-induced apoptosis, and prevented ischemia-induced angiogenesis and hypercholesterolemia-induced ECs dysfunction in mice [65–68]. Loss of TrxR2 increased mtROS and impaired mitochondrial membrane potential, angiogenesis and arteriogenesis, resulting in a pro-inflammatory vascular phenotype [69]. All these results indicated that Trx2 plays a critical role in ECs function and angiogenesis by decreasing ROS, apoptosis and inflammation, and increasing NO availability. In addition, mitochondrial interactome of Trx2 showed that Trx2 was probably involved in mitochondrial integrity, formation of iron sulfur clusters, detoxification of aldehydes, protein synthesis, folding, ADP ribosylation, amino acid and lipid metabolism, glycolysis, TCA cycle and electron transfer chain [70]. Thus, further validation of these putative functions of Trx2 will provide potential therapeutic strategy for ECs dysfunction and vascular diseases.

### Isocitrate dehydrogenase 2 (IDH2)

Cytoplasmic IDH1 and mitochondrial matrix IDH2 are NADP^+^-dependent enzymes that catalyze the oxidative decarboxylation of isocitrate to produce α-ketoglutarate and NADPH, playing key roles in the TCA cycle, redox status and cellular homoeostasis. Both IDH1 and IDH2 mutations have been reported in several cancers such as acute myeloid leukaemia, chondrosarcoma and glioma [71]. The mechanism responsible for tumor angiogenesis and growth is that IDH mutation-induced 2-hydroxyglutarate production caused histone and DNA methylation, which repressed gene expression, increased HIF transcription, promoted mitochondrial energy metabolism and ECs migration through SLC1A1-mediated transportation [71, 72]. IDH2 deficiency in dermal fibroblasts led to increased apoptosis through ROS-dependent ATM-mediated p53, decreased proliferation, migration, invasion, VEGF expression and extracellular matrix protein fibronectin, resulting in delayed wound healing [73]. In HUVECs, IDH2 knockdown decreased the expression of mitochondrial complex I, III and IV, induced endothelial dysfunction and pro-inflammatory cytokines via p66shc-mediated mitochondrial oxidative stress [74, 75]. These results suggested that IDH2 is required for ECs angiogenesis through improving mitochondrial function. Furthermore, the competitive inhibitor of IDH1 and IDH2, oxalomalate, inhibited HIF-1α-mediated VEGF expression through ROS-controlled E2F1 activity in retinal pigment epithelium cells and decreased the development of choroidal neovascularization in the mouse model of age-related macular degeneration, providing a novel therapeutic strategy for treating pathological angiogenesis through IDH inhibition [76].

## Mitochondrial Ca^2+^ in ECs angiogenesis

**Fig. 2:**
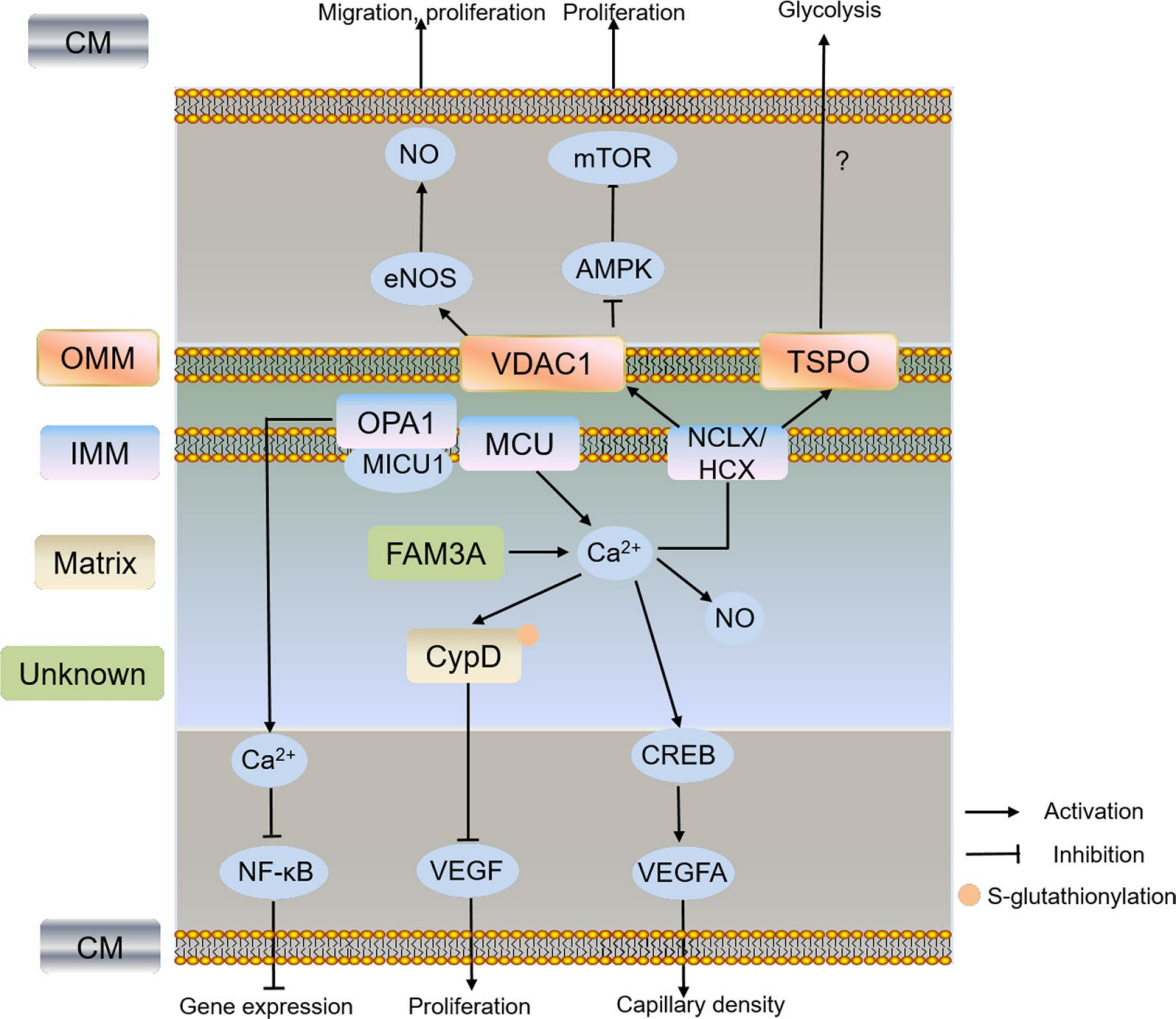
Schematic pathway of the mitochondrial Ca^2+^ and related protein in regulating ECs function and angiogenesis. Different colors of boxes indicate different locations of protein in cells listed on the left. VDAC1 promoted ECs migration and proliferation through inhibition of AMPK-mTOR signaling or eNOS/NO pathways. FAM3A increased angiogenesis by enhancing (CREB)-dependent VEGFA transcription through ATP/P2 receptor/Ca^2+^ pathway. Depletion of CypD increased Ca^2+^ and NADH levels, promoted VEGF-induced proliferation and angiogenesis, accelerated wound healing and neovascularization, while S-glutathionylation of CypD-C203 is associated with mitochondrial O_2_^−^ production. VDAC1: voltage-dependent anion channel 1; TSPO: translocator protein; CypD: cyclophilin D; MCU: mitochondrial calcium uniporter; NCLX: Na^+^/Ca^2+^ exchanger; HCX: H^+^/Ca^2+^ exchanger; OPA1: optic atrophy 1 protein 1; CM: cell membrane; OMM: outer mitochondrial membrane; IMM: inner mitochondrial membrane

Ca^2+^ has been recognized as a second messenger that regulates a series of cellular processes such as gene transcription, cell proliferation, differentiation and death. The increase of intracellular Ca^2+^ levels and multiple Ca^2+^ signatures (transients, biphase and oscillations) induced by VEGF is recognized as a key pro-angiogenic signaling that increases ECs proliferation, migration and tube formation [77, 78]. Several mechanisms regulate the process of Ca^2+^ release including phospholipase C-γ(PLCγ)-InsP3 pathways, store-operated Ca^2+^ entry (SOCE) channel [79], nicotinic acid adenine-dinucleotide phosphate (NAADP)/two-pore channel 2 (TPC2) [80] and ROS-transient receptor potential melastatin-2 (TRPM2) [81]. Of note, 75% of intracellular Ca^2+^ in ECs were stored in endoplasmic reticulum (ER), while the remaining 25% were stored in the mitochondria. Mitochondrial Ca^2+^ was reported to act as regulators and buffer of intracellular calcium signaling and homeostasis, which plays an essential role in dehydrogenases activation, ATP production and cell fate [82]. The content of mitochondrial Ca^2+^ is tightly regulated by the influx protein mitochondrial Ca^2+^ uniporter complex (MCUC), and efflux proteins NCLX and H^+^/Ca^2+^ exchanger (HCX). MCUC is composed of pore-forming subunit mitochondrial calcium uniporter (MCU), and their regulators MCUb, mitochondrial calcium uptakes (MICUs), EMRE and MCUR1. MCU is a mitochondrial luminal redox sensor and S-glutathionylation of MCU Cys-97 by hypoxia promoted MCUC assembly and induced mitochondrial matrix Ca^2+^ uptake, sensed mtROS and sensitized cells to death [83]. MCU was required for the promotion of metastasis and ECs angiogenesis through negative sorting of miR-4488 in extracellular vesicles (EVs), which directly targeted CX3CL1 in breast cancer [84]. Inhibition of MCU decreased Ca^2+^-dependent mitochondrial NO production in bovine vascular ECs (BVECs) [85], but whether these proteins participate in physiological relevance such as angiogenesis is still unknown. Ca^2+^ may also escape the mitochondrial matrix through opening of mitochondrial permeability transition pore (mPTP) complex, which consists of VDAC, TSPO and CpyD proteins. In addition, transfer of Ca^2+^ in mitochondria-endoplasmic reticulum contact sites in response to external stimulation via inositol 1,4,5-trisphosphate (IP3) receptor channels (IP3Rs) also provides platforms in regulation of mitochondrial Ca^2+^. Herein, the mPTP components and the mitochondrial proteins that are involved in mitochondrial Ca^2+^ homeostasis and regulating ECs angiogenesis were reviewed (Fig. 2).

### Voltage-dependent anion channel 1 (VDAC1)

Three isoforms of outer mitochondrial membrane (OMM) protein VDACs, VDAC1, VDAC2 and VDAC3, have been identified so far [86]. VDACs regulate the exchange of calcium and metabolites including ATP, ADP, NADH and pyruvate between cytosol and mitochondria. Furthermore, VDACs could act as a metabolic switch to regulate mitochondrial metabolism and aerobic glycolysis in tumor depending on the magnitude and duration of VDAC opening [87]. VDAC1 is a component of the mPTP that regulates cell proliferation, apoptosis, mitochondrial metabolism and PTEN-induced putative protein kinase 1 (PINK1)/Parkin-mediated mitophagy [88–90]. Both VDAC1 and VDAC2 were reported to bind eNOS, and depletion of VDAC2 but not VDAC1, blocked the histamine-induced eNOS/NO activity in human pulmonary artery ECs (HPAECs), indicating VDAC2 is mainly responsible for pulmonary circulation [91]. VDAC1 was found to be a novel upstream regulator of mTOR signaling and plays a critical role in ECs proliferation. VDAC1 knockdown or inhibition by erastin and itraconazole decreased mitochondrial ATP production and increased the AMP:ATP ratio, which in turn activated AMPK and phosphorylated raptor, inhibited mTOR activity and ECs proliferation in HUVECs, suggesting that targeting VDAC1 is an effective way to inhibit ECs angiogenesis [92].

### Cyclophilin D (CypD)

CypD is one of the well-characterized mPTP complex involved in Ca^2+^ levels, energy metabolism, apoptosis, autophagy and programmed necrosis [93–95]. The mPTP opening is regulated by mitochondrial phosphate carrier-CypD interaction and mitochondrial ATP synthase inhibitory factor 1-p53-CypD complex, which results in loss of membrane potential, uncoupling of oxidative phosphorylation, depletion of ATP, and increase of ROS [96, 97]. Binding of CypD to mitochondrial signal transducer and activator of transcription 3 (STAT3) is responsible for reducing oxidative stress-induced mtROS production, while depletion of CypD promoted normal and tumor cell proliferation, migration and cell invasion through phosphorylation of STAT3 Tyr-705 [98, 99]. In HAECs, S-glutathionylation of CypD-C203 induced by angiotensin II is associated with mitochondrial O_2_^−^ production and oxidative stress, while depletion of CypD increased Ca^2+^ and NADH levels, promoted VEGF-induced proliferation and angiogenesis. Furthermore, knockout of CypD in mice or inhibition of CypD with sanglifehrin A increased mitochondrial protein acetylation and metabolism, accelerated wound healing and neovascularization, lowered blood pressure and improved vascular relaxation [100–102]. These results indicated that targeting CypD is effective to promote ECs angiogenesis and wound healing, and improve vascular relaxation. Besides S-glutathionylation, other post-translational modifications also activated mPTP and increased cardiomyocyte death. For example, phosphorylation of CypD-S191 is required for mPTP opening by regulation of its binding to mPTP core component oligomycin sensitivity conferring protein (OSCP) [103]. Phosphorylation of CypD-S42 in MCU-KO mice and acetylation of CypD by SIRT3 under ischemia–reperfusion also activated mPTP [104, 105]. In contrast, s-nitrosation [106] and de-acylation of CypD [107] did not induce mPTP opening and protected cardiomyocytes from necrosis. Thus, exploration of the specific modifications responsible for CypD function in relation to ECs angiogenesis is important and worthy.

### Translocator protein (TSPO)

TSPO is a 18 kDa OMM protein and is up-regulated under various pathological conditions such as cancer, neurological diseases and nonalcoholic fatty liver disease [108, 109]. TSPO is a cholesterol-binding protein required for cholesterol import into mitochondria, which is necessary for preimplantation embryo development, steroid biosynthesis, energy balance and bile acid synthesis [109–111]. In addition, the allosteric regulation of TSPO homo- and hetero-oligomerization is dependent on cholesterol-binding, thus affecting mitochondrial function [112]. Although TSPO depletion in cells showed changes in ROS production, Ca^2+^ signaling, autophagy, cholesterol efflux, and mitochondrial oxygen consumption rate (OCR), the precise function of TSPO in processes such as steroidogenesis, heme biosynthesis and mPTP component remains to be studied [113]. Recent studies revealed that a multimolecular complex formed by TSPO, acyl-CoA binding domain containing 3 (ACBD3) and protein kinase A (PKA) controlled intracellular Ca^2+^ dynamics, redox balance, and coupled pro-survival retrograde response between mitochondria and nucleus [114, 115]. Reactive phagocytes play important roles in etiology of age-related macular degeneration (AMD). TSPO is a key regulator of NOX1-dependent ROS overproduction by increase of cytosolic Ca^2+^ in the retina phagocyte. TSPO knockout or specific ligand XBD173 dampened phagocyte reactivity towards a neuroprotective phenotype, decreased retina pro-angiogenesis and vascular leakage, limiting pathological choroidal neovascularization [116]. TSPO was upregulated in glioblastomas-derived vascular ECs. Knockout of TSPO in glioma cell increased glioma growth and angiogenesis by promoting glycolysis and reducing oxidative phosphorylation, indicating its role in controlling the metabolic balance during tumor growth and angiogenesis. Furthermore, TSPO antibody-based therapy inhibited tumor growth, decreased vascular permeability and provided effective therapy against glioblastomas [117, 118]. These results indicated that TSPO is an important therapeutic target for angiogenesis under different pathological conditions. However, our knowledge on the role and mechanism of TSPO in physiological ECs angiogenesis and metabolism is scarce and further studies are needed.

### FAM3A

FAM3A is one of the cytokine-like proteins family including FAM3A, FAM3B, FAM3C, and FAM3D. FAM3A was previously reported to attenuate hyperglycemia, insulin resistance, gluconeogenesis and lipogenesis, and activate PI3K-Akt signaling pathways by promotion of Ca^2+^/calmodulin in liver, which was similar to FAM3B [119]. FAM3A protein is a novel mitochondrial protein highly expressed in vascular endothelium that regulates mitochondrial respiratory activity and ATP production. Overexpression of FAM3A can increase capillary density and angiogenesis by enhancing cAMP response element binding protein (CREB)-dependent VEGFA transcription through ATP/P2 receptor/Ca^2+^ pathway in HUVECs, indicating upregulation of FAM3A is effective as a pro-angiogenic therapy [120].

## Mitochondrial H_2_S in ECs angiogenesis

**Fig. 3:**
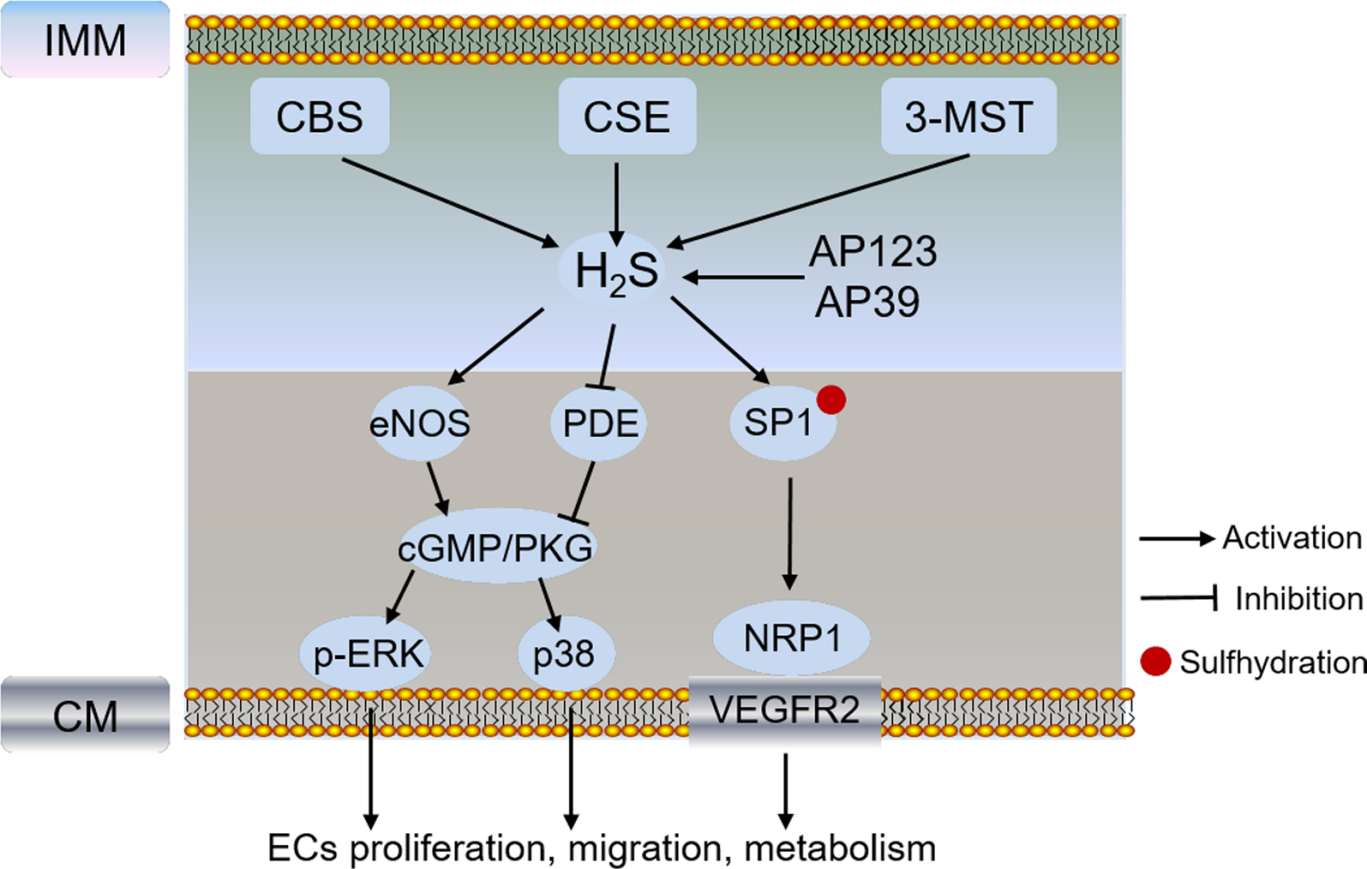
Schematic pathway of the mitochondrial H_2_S and related protein in regulating ECs function and angiogenesis. Different colors of boxes indicate different locations of protein in cells listed on the left. H_2_S promoted angiogenesis by increasing cGMP production through activating eNOS/NO and prevention of cGMP breakdown through inhibiting phosphodiesterase (PDE), thus triggering cGMP/PKG-dependent downstream signaling such as ERK1/2 and p38 in case of angiogenesis. Silencing of CSE exacerbated mtROS production and decreased eNOS-NO production. Silencing CBS decreased transcription of VEGFR2 and neuropilin 1 (NRP1) by sulfhydration of specificity protein 1 (Sp1) at Cys68 and Cys755. Knockdown of 3-MST in ECs reduced VEGF-induced cell proliferation, migration, and tube-like network formation, inhibited mitochondrial oxidative phosphorylation. CBS: cystathionine beta-synthase; CSE: cystathionine-gamma-lyase; 3-MST: 3-mercaptopyruvate sulfurtransferase; SP1: specificity protein 1; PDE: phosphodiesterase; cGMP/PKG: cyclic guanosine 5′-monophosphate/protein kinase G; NRP1: neuropilin 1; CM: cell membrane; IMM: inner mitochondrial membrane

H_2_S is a water-soluble and oil-soluble gasotransmitter that easily crosses plasma membranes, regulating a series of physiological processes such as vascular tone, endothelial angiogenesis, mitochondrial function and inflammation [121, 122]. H_2_S was reported to promote angiogenesis by facilitation of cGMP production through activating eNOS/NO and prevention of cGMP breakdown through inhibiting phosphodiesterase (PDE), thus triggering cGMP/PKG-dependent downstream signaling such as ERK1/2 and p38 in case of angiogenesis [123]. The major enzymes responsible for intracellular H_2_S production are cystathionine beta-synthase (CBS), cystathionine-gamma-lyase (CSE), 3-mercaptopyruvate sulfurtransferase (3-MST) and cysteine aminotransferase [124]. CSE is localized only in the cytosol and could translocate to mitochondria under specific stimulations, while CBS and 3-MST are localized in both mitochondria and cytoplasm [125–127]. Although the enzymes promote mitochondrial H_2_S production and regulate energy production, the mechanism responsible for ECs function and angiogenesis are different (Fig. 3). Overexpression of CSE in bEnd3 microvascular ECs (MECs) attenuated high glucose-induced mtROS production, while silencing of CSE exacerbated this response and decreased eNOS-NO production [128, 129]. Silencing CBS in ECs decreased transcription of VEGFR2 and neuropilin 1 (NRP1) by sulfhydration of specificity protein 1 (Sp1) at Cys68 and Cys755, indicating CBS-mediated protein sulfhydration maintains vascular health and function [130]. Knockdown of 3-MST in ECs reduced VEGF-induced cell proliferation, migration, and tube-like network formation, inhibited mitochondrial oxidative phosphorylation, increased glucose uptake and perturbed ECs metabolism [127]. Furthermore, mitochondrial-targeted H_2_S donor AP123 and AP39 were reported to improve respiratory complex II/III activity and inhibit mitochondrial oxidant production, protecting high glucose and hypoxia-induced ECs damage and trophoblasts anti-angiogenesis [131, 132]. These indicate that targeting H_2_S to mitochondria is a protective strategy to prevent stress-induced endothelial function and angiogenesis.

Of note, H_2_S plays dual roles in regulating mitochondrial metabolism. It improves mitochondrial function at low concentrations, while inhibits it at high concentrations. Mitochondrial H_2_S donates electrons to the mitochondrial electron transport chain via sulfide:quinone oxidoreductase (SQR) to maintain mitochondrial electron flow and energetics [133]. However, when complex IV was inhibited under high H_2_S concentrations, complex II sustained SQR-dependent H_2_S clearance by using fumarate as an electron acceptor [134], indicating different metabolic mechanisms of H_2_S existed in mitochondria under specific condition. Indeed, the metabolic enzymes of H_2_S such as SQR, persulfide dioxygenase and sulfite oxidase are also localized in mitochondria. The rate of mitochondrial H_2_S oxidation and metabolism depends on H_2_S concentrations, NADH pool and oxygen pressure [135]. Whether these enzymes are involved in ECs angiogenesis and the potential mechanism need further investigation.

## Mitophagy

**Fig. 4:**
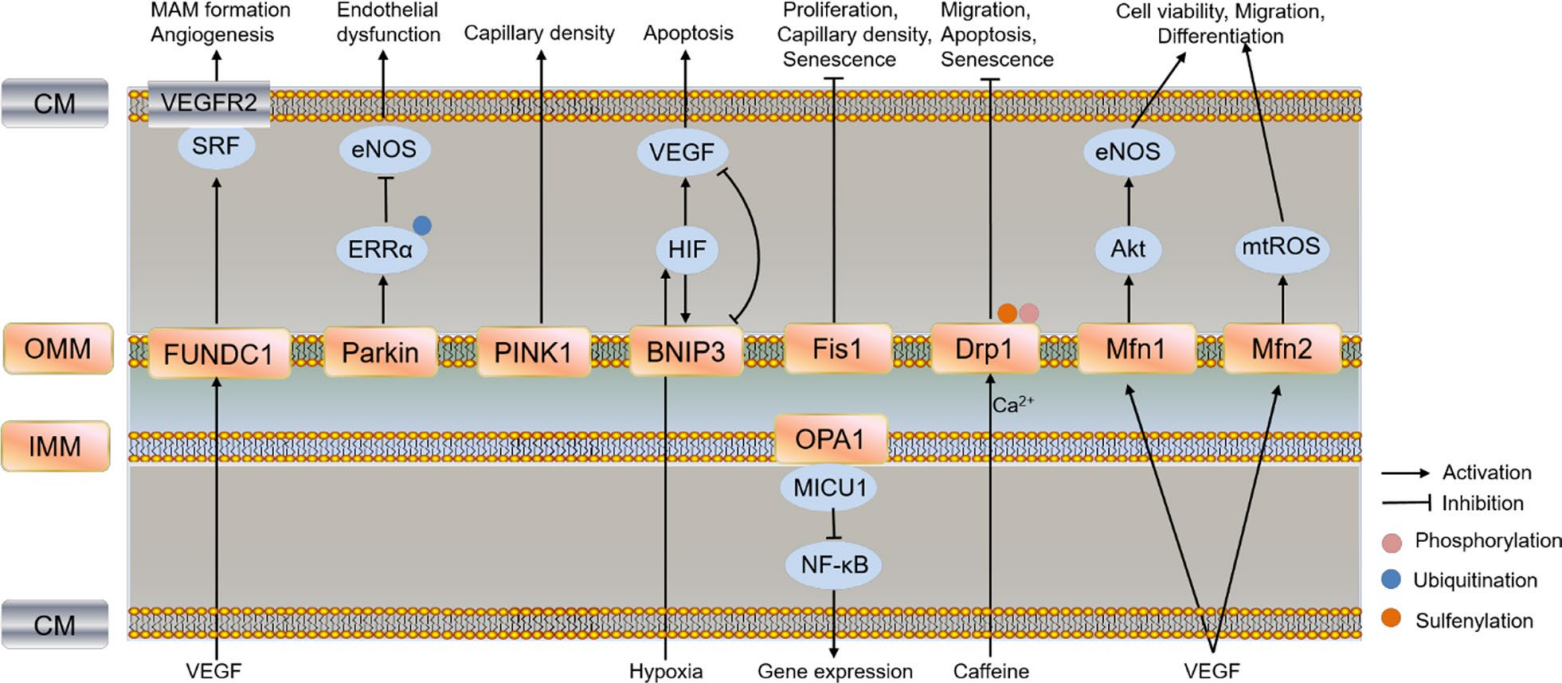
Schematic pathway of mitophagy and mitochondrial dynamics (including fusion and fission) in regulating ECs function and angiogenesis. Different colors of boxes indicate different locations of protein in cells listed on the left. PINK1 knockout impaired mitochondrial function, reduced cardiac capillary density and angiogenesis in heart; Parkin decreased eNOS expression and induced mitochondrial dysfunction by ubiquitination of ERRα; Deletion of FUNDC1 disrupted MAM formation and angiogenesis through decreasing binding of SRF to VEGFR2, and decreased VEGFR2 production; HIF-1-induced VEGF and BNIP3 regulated the balance between survival and apoptosis; Knockdown of Mfn2 led to mtROS production and mitochondrial dysfunction, and disrupted mitochondria-ER contact sites, while knockdown of Mfn1 reduced VEGF-induced Akt-eNOS signaling in HUVECs; Silencing Drp1 suppressed caffeine-induced lamellipodia formation and migration, while overexpression of Drp1 improved angiogenic function, and prevented apoptosis via mitochondrial Ca^2+^ dependent. Sulfenylation of Drp1 Cys644 and phosphorylation regulated ECs senescence; Fis1 increased proliferation, capillary density and angiogenesis, restored senescence phenotype in senescent EPCs. FUNDC1: FUN14 domain-containing 1; Parkin: E3 ubiquitin-protein ligase parkin; BNIP3: Bcl-2 nineteen-kilodalton interacting protein 3; Fis1: fission 1; Drp1: dynamin-related protein 1; Mfn1/2: mitofusins 1/2; OPA1: optic atrophy 1 protein 1; ERRα: estrogen-related receptor α; CM: cell membrane; OMM: outer mitochondrial membrane; IMM: inner mitochondrial membrane

Autophagy is an evolutionarily conserved process essential for elimination of dysfunctional organelles and components, and plays an important role in maintaining ECs proliferation, migration and permeability under stress conditions. For example, endothelial ATG5-deficient cells displayed impaired mitochondrial function, decreased mtROS production and VEGFR2 phosphorylation under hypoxia/reoxygenation, while overexpression of ATG5 increased BAECs tube formation and migration [136, 137]. Although Atg7 deletion by using the Amhr2-Cre-driven model was confirmed by autophagic deficit in uterine stromal, myometrial, and vascular smooth muscle cells, but not in ECs, the model showed dilation of blood vessels, reduced endothelial junction-related proteins, increased vascular permeability, and increased VEGFA and NOS1 expression, indicating autophagy defect in other cell types also regulate ECs function and vascular development [138]. The specific mechanism of autophagy in regulating endothelial lineage and various stem cells angiogenesis has been reviewed, including increasing the resistance to cell death, adaptation to hypoxic conditions, and sustaining the high energy demand [139]. Mitophagy is mainly responsible for elimination of dysfunctional mitochondria and ROS production, and maintenance of mitochondrial quality. In this review, we will describe the molecular mechanisms underlying major mitophagy protein-mediating angiogenesis in ECs (Fig. 4).

### PINK1/Parkin

In healthy mitochondria, the serine/thereonine kinase PINK1 is imported by TIM/TOM complex into the IMM and then hydrolyzed by multiple proteases. Upon mitochondrial depolarization, accumulation of PINK1 on OMM serves a sensor for mitochondrial damage, recruits E3 ubiquitin ligase Parkin and promotes ubiquitination of mitochondria protein for autophagosome engulfment [140, 141]. Indeed, PINK1 is not only involved in promoting mitophagy and maintaining mitochondrial function, early studies also reported that increased expression of PINK1/BRPK in cancer cells was associated with higher metastatic potential and invasion [142, 143], suggesting PINK1 may promote tumor metastasis and angiogenesis. PINK1 knockout mice showed increased oxidative stress, impaired mitochondrial function, reduced cardiac capillary density and angiogenesis in heart [144]. Our recent studies suggested that knockout of PINK1 in porcine trophectoderm cell decreased protein expression of cathepsin B, ALDH2, tumor necrosis factor receptor superfamily member 12A, heat shock protein beta-1 and increased CD63 expression, which are involved in ECs angiogenesis (unpublished data). Although PINK1 is not involved superoxide production in BAECs under acute hypoxia, whether PINK1 promotes ECs angiogenesis is unknown, and the potential mechanism remains to be further studied [30]. Unlike PINK1, the downstream Parkin has been reported to inhibit tumor angiogenesis through ubiquitination degradation of HIF-1α at lysine 477 [145], and inhibiting JAK2/STAT3/VEGF pathways in osteosarcoma [146]. In mouse aortic ECs (MAECs), overexpression of Parkin decreased eNOS expression and induced mitochondrial dysfunction by ubiquitination of estrogen-related receptor α (ERRα), independent of autophagy and apoptosis, indicating activation of parkin in ECs might be detrimental in ECs [147]. These results suggested PINK1 and Parkin possiblely have opposite effect on EC angiogenesis, and the specific mechanisms need further study.

### FUN14 domain-containing 1 (FUNDC1)

OMM protein FUNDC1 functions as a mitophagy receptor, which interacts with LC3 through an LC3-interacting region to promote mitophagy [148]. The FUNDC1-mediated mitophagy is also activated under dephosphorylation at Ser13 by PGAM5 and inhibited under ubiquitylation at Lys119 by MARCH5 in response to hypoxia [149, 150]. Besides mitophagy, FUNDC1 also participates in mitochondria-associated endoplasmic reticulum membranes (MAM) formation, Ca^2+^ homeostasis, mitochondrial dynamics and insulin resistance [151–154]. FUNDC1-dependent MAM promoted breast cancer proliferation and migration through activating cytoplasmic Ca^2+^-NFATC1-BMI1 axis, which is also required for VEGF-induced angiogenesis [155]. Furthermore, ECs specific deletion of FUNDC1 disrupted VEGF-induced MAM formation and angiogenesis through decreasing the binding of serum response factor (SRF) to VEGFR2, resulting in decreased VEGFR2 production. In addition, a 12 amino acids peptide blocking FUNDC1 and IP3R1 delayed endothelial spheroid-sprouting and vessel density since IP3Rs is responsible for Ca^2+^ release from the ER and MAM formation [154]. These results indicated FUNDC1-mediated MAM is a promising target for treating tumor and angiogenesis-related disorders.

### Bcl-2 nineteen-kilodalton interacting protein 3 (BNIP3)

OMM-localized receptor BNIP3, one of the Bcl-2 homology domain (BH3)-only Bcl-2 family, is involved in mitophagy, necrosis, apoptosis, immunity, metabolic homeostasis and zonation in favor of glycolysis, glutamine synthesis, lipogenesis rather than gluconeogenesis, urea cycle, fatty acid oxidation [156–158]. BNIP3 showed differential expression and localization (mitochondria, cytoplasm and nucleus) in various cell types under different stress, which is regulated at both transcriptional and post-transcriptional level [158–161]. Production of mtROS caused by loss of BNIP3 induced cell proliferation and tumor growth, increased gene expression of HIF1α-dependent glycolysis and angiogenesis, suppressed oxidative phosphorylation, suggesting BNIP3 plays an inhibitory role in mammary tumorigenesis [162]. HIF-1-induced VEGF and BNIP3 regulated the balance between survival and apoptosis in ECs. BNIP3 knockdown reduced ECs apoptosis in VEGF knockdown under hypoxic condition, suggesting VEGF and BNIP3 have antagonistic effect on ECs apoptosis [163]. However, the role and mechanism of BNIP3 in regulating ECs angiogenesis remains further investigation.

## Fusion and Fission

Mitochondria are highly dynamic structures that adjust its shape and distribution depending on the equilibrium of two opposing processes, fission and fusion. Mitofusins (Mfn1/2) and optic atrophy 1 protein (OPA1) are required for mitochondrial fusion. Inner membrane protein OPA1 is required for tumor vascularization, metastasis and growth, and ECs angiogenesis by maintaining cytosolic Ca^2+^ buffering through interacting with MICU1 and inhibiting NF-κB, which activated the pro-angiogenic gene expression (Fig. 4). Furthermore, first-in-class OPA1 inhibitor MYLS22 limits tumor growth and metastatization, indicating target Opa1 is effective for inhibiting tumor angiogenesis [164]. However, studies about Mfns regulating ECs angiogenesis are conflicting and need further investigation. Mfns knockdown induced HUVECs apoptosis and mitochondrial fragmentation but did not affect migration, proliferation, and tubulogenesis [164]. Other researchers suggested Mfns was responsible for VEGF-mediated migration and differentiation in HUVECs and vascular remodeling in astrocytes. Knockdown of Mfn2 led to mtROS production and mitochondrial dysfunction in HUVECs, and disrupted mitochondria-ER contact sites in astrocytes, while knockdown of Mfn1 reduced VEGF-induced Akt-eNOS signaling in HUVECs, indicating the different mechanisms of Mfn1/2 are required for ECs function and survive [165, 166].

Dynamin-related protein (Drp1) and fission 1 (Fis1) are required for mitochondrial fission. Drp1 accumulates from the cytosol to the OMM that promotes fission in GTP-dependent manner. Silencing Drp1 suppressed caffeine-induced lamellipodia formation and hypoxia-induced ECs tube formation and PAECs migration through MCU-mediated mitochondrial Ca^2+^, while overexpression of Drp1 improved angiogenic function, and prevented apoptosis via mitochondrial Ca^2+^ dependent pathways in different ECs [167–169]. Furthermore, a number of studies reported that post-translational modifications of Drp1 such as sulfenylation of Cys644 by loss of protein disulfifide isomerase (PDI) also induced ECs senescence and impaired wound healing, while Drp1-C644A improved wound healing and angiogenesis in PDIA1 deficient mice [170]. Inhibition of Drp1 phosphorylation at Ser616 by Bax inhibitor 1 (BI1)-mediated inactivation of Syk-NOX2 pathways abolished fatal mitochondrial fission and preserving cardiac microvascular IR injury [171]. Similar to Drp1, enhanced expression of Fis1 increased proliferation, capillary density and angiogenesis, restored senescence phenotype in senescent EPCs since senescent EPCs showed deteriorated therapeutic angiogenesis and decreased Fis1 expression [172]. The above results indicated that induction of mitochondrial fussion and fission promoted ECs function and angiogenesis.

## Other mitochondrial protein in ECs angiogenesis

### Prohibitin1 (PHB1)

The highly conserved prohibitins (PHB1 and PHB2) belong to the SPFH-family members and function as scaffold proteins and membrane organizers. The IMM protein PHB1 binds to PHB2 to form heterodimers and regulates cellular senescence, apoptosis, assembly of mitochondrial respiratory chain, maintenance of mitochondrial cristae junctions and mitochondrial biogenesis [173, 174] Furthermore, the PHB colocalized with annexin A2 in the vascular ECs to regulate CD36-mediated fatty acid transport, and targeting a proapoptotic peptide to PHBs reversed obesity without any adverse effect [175, 176], indicating the potential for development of targeted drugs for treatment of obesity-related metabolic diseases. PHB1 was downregulated in aging and inflammatory bowel diseases, but upregulated in high metastatic cancer cells [177–179]. Knockdown of PHB1 in ECs resulted in mitochondrial dysfunction and ROS overproduction via inhibition of complex I, cytoskeletal rearrangements and cell senescence by increasing Akt and cytoskeletal protein Rac1, resulting in reduction of ECs migration and tube formation in vitro, and formation of functional blood vessels in vivo [180]. Activation of prohibitins by NAD^+^ precursor nicotinamide riboside ameliorated TGF-β-induced endothelial-to-mesenchymal transition (EndoMT), delayed senescence and increased mouse life span [181, 182]. These studies suggested induction of PHBs is beneficial for promotion of angiogenesis by maintaining mitochondrial integrity and inhibiting senescence.

### Uncoupling protein 2 (UCP2)

IMM proteins UCPs (UCP1 to UCP5) are a subfamily of the mitochondrial carrier family that uncouple proton flux and mediate transport of small molecules metabolites such as oxaloacetate, Cl^−^, and H^+^. Unlike UCP1 (expressed in brown adipocytes) and UCP3 (expressed in skeletal muscle), UCP2 is expressed in several tissues including liver, brain, heart, spleen and kidney, and regulates mitochondrial antioxidant defense, glucose and lipid metabolism, insulin secretion and angiogenesis [183–185]. Overexpression of UCP2 reversed tube formation through blocking mitochondrial superoxide production and maintaining high levels of NO in MAECs of AMPKα1^−/−^ or AMPKα2^−/−^ mice [186]. Similarly, UCP2 normalized the nitrite/nitrate concentration in serum, promoting blood pressure in kidney of DJ-1^−/−^ mice [187]. Loss of UCP2 in BAECs induced mitochondrial network fragmentation and premature senescence through superoxide-mediated p53 activation [188]. These results showed that UCP2 promoted angiogenesis, blood pressure and improved endothelial function though improvement of mitochondrial function and inhibition of mtROS production. Indeed, superoxide could induce UCP2 expression, and elevated UCP2 in turn decreases ROS production, indicating a negative feedback regulation. But the precise regulatory mechanism between superoxide and UCP2 remains further study. Of note, knockdown of UCP2 or treatment with UCP2 inhibitor genipin significantly increased glucose transporter type 1-dependent glycolysis, VEGF-induced VEGFR2 phosphorylation and cell proliferation in human retinal microvascular ECs (HRMECs) and reduced intravitreal neovascularization in rats [189]. The results indicated different regulatory role of UCP2 in endothelial function and angiogenesis under specific condition. On the other hand, UCP2 positively promoted glycolysis, mitochondrial fatty acid oxidation and decreased mitochondrial pyruvate catabolism in pluripotent stem cells, leukemia cells, embryonic fibroblasts and cancer cells, which are characterized by high proliferation rate [190–193]. These results suggested that UCP2 appeared as an excellent candidate for linking energy metabolic switch and cell proliferation. Thus, understanding the tissue-specific role and mechanism of UCP2 in regulating metabolic characteristics, proliferation and angiogenesis in ECs is of great importance.

### Sirtuins (SIRTs)

The mammalian class III NAD^+^-dependent histone deacetylases SIRTs family proteins, SIRT1 to SIRT7, have been identified. Among these, SIRT1 is expressed in the nucleus and cytoplasm, and can translocate to mitochondria; SIRT2 is expressed in the cytoplasm; SIRT3, SIRT4 and SITR5 are located in mitochondrial matrix, and SIRT6 and SIRT7 are mainly located in the nucleus. These SIRTs are responsible for controlling important cellular processes, including cellular metabolism, antioxidant defense, mitochondrial homeostasis and inflammation in response to nutrient stress and membrane depolarization. Depletion of SIRTs has been linked to several pathologies such as insulin resistance, cardiovascular disease and aging [194]. Herein, the roles and potential mechanism of mitochondrial SIRT3, SIRT4 and SITR5 in ECs function and angiogenesis were reviewed and discussed.

SIRT3 has the strongest deacetylase activity among the SIRTs and is the only member linked to the longevity. SIRT3 has been reported to control the activities of metabolic enzyme and components of oxidative phosphorylation, such as regulation of mitochondrial redox status through deacetylation of IDH2 [195], oxidative phosphorylation through deacetylation of NADH dehydrogenase (ubiquinone) 1 alpha subcomplex subunit 9 (NDUFA9) [196], ketogenesis through deacetylation of 3-hydroxy-3-methylglutaryl CoA synthase 2 (HMGCS2) [197], urea cycle through deacetylation of ornithine transcarbamoylase (OTC) [198], fatty acid oxidation through deacetylation of long-chain acyl coenzyme A dehydrogenase (LCAD) [199], which is tissue and environment dependent [200–202]. SIRT3 acted as a tumor suppressor gene and its downregulation promoted tumor cell migration and metastasis [203]. In ECs, overexpression of SIRT3 improved oxidative injury and EPCs reendothelialization capacity [204], EndoMT [205], high glucose and insulin-induced retinal neovascularization [206], cardiac remodeling and lymphangiogenesis [207, 208], diastolic dysfunction [209] and decreased CR6 interacting factor 1 (CRIF1) deficiency-induced premature senescence [210]. The mechanism responsible for ECs angiogenesis includes deacetylation of MnSOD and decreasing mtROS production [204], decreasing vascular permeability and inflammation [211], metabolic reprogramming through regulation of 6-phosphofructo-2-kinase/fructose-2, 6-bisphosphatase 3 (PFKFB3) [209], VEGFC/VEGFR3 and ERK pathways [207], autophagy-dependent degradation of pyruvate kinase M2 (PKM2) [205] and promotion of PINK1/Parkin mitophagy [208], indicating diverse mechanisms of regulating ECs function and angiogenesis by SIRT3.

Unlike SIRT3, SIRT4 is a mitochondrial ADP-ribosyltransferase that acts as a “guardian of cellular metabolism” through repression of pyruvate dehydrogenase complex activity and malonyl CoA decarboxylase, which are responsible for production of acetyl CoA from pyruvate and malonyl CoA, respectively [212, 213]. SIRT4 was also reported as a tumor suppressor or promoting protein in different tumors, involving in tumor cell proliferation, migration, invasion and metastasis [214, 215]. In HUVECs, SIRT4 knockdown increased the expression of pro-inflammatory cytokines, MMP-9 and adhesion molecule ICAM-1, which are involved in inflammation, vascular remodeling and angiogenesis [216]. However, the potential mechanism of SIRT4 in angiogenic signaling is unknown and needs further study.

SIRT5 is a NAD^+^-dependent protein that has strong desuccinylase, demalonylase, deglutarylase activities and weak deacetylase activity. It regulates mitochondrial fatty acid oxidation through desuccinylation of ECHA [217], urea cycle through deacetylation of carbamoyl phosphate synthetase 1 [218], glycolysis through malonylation of GAPDH activity [219] and ketogenesis through succinylation of the HMGCS2 [220]. CXCR4/JAK2/SIRT5 pathways were reported to regulate mitochondrial function of late EPCs, which is required for capillary formation, angiogenic capacity and wound healing [221, 222]. Knockdown of SIRT5 decreased endothelial permeability and upregulated tight junction proteins via PI3K/Akt pathway in human brain microvascular ECs (HBMECs) after exposure to hypoxia and reoxygenation (H/R) [223], suggesting SIRT5 may serve as a novel therapeutic target for repair ECs damage in brain.

The above results suggested specific mechanisms of mitochondrial SIRTs in the regulation of ECs angiogenesis. However, since mitochondrial SIRT3-5 are mainly involved in metabolic regulation, how these SIRTs-mediated metabolites and signaling molecules are involved in ECs function and angiogenic signaling cascade is unknown and requires further study.

### Cytochrome P450 1B1 (Cyp1B1)

Cytochrome P450 (Cyp) enzymes are heme containing monooxygenases that are involved in metabolism of endogenous substrates such as steroids and xenobiotics, depending on oxygen and NADPH levels. The major Cyp enzymes are localized in endoplasmic reticulum and microsome membrane, such as Cyp2 (2 J, 2B, 2C) and Cyp4A family, which regulate vascular functions, EC angiogenesis and tumor metastasis [224]. Mitochondrial P450 families (Cyp1, Cyp11, Cyp24 and Cyp27) have been found in animals but not in plant and fungi, suggesting the biological evolution of eukaryotic organisms [225]. Cyp1B1 is an unusual member of Cyp1 family proteins (Cyp1A1, Cyp1A2, and Cyp1B1) that metabolizes endogenous and exogenous substrates such as estrogen, melatonin, dietary flavanoids, and regulates iron levels, redox homeostasis, mitochondrial dysfunction and angiogenesis [226, 227]. Vascularization is a highly orchestrated process that depends on complex coordination of perivascular supporting cells, astrocytes and ECs according to their anatomical location [228]. Cyp1B1 deficient mice showed increased proliferation, migration, and adhesion in retinal astrocytes and perivascular supporting cells, while impaired revascularization, decreased endothelial NO synthase and migration, increased oxidative stress and expression of antiangiogenic factor thrombospondin-2 (TSP2) in retinal ECs [229–231]. Furthermore, Cyp1B1 deletion in pericytes resulted in attenuation of retinal neovascularization but not in other cell types such as ECs, astrocytes, and trabecular meshwork ECs, indicating the roles of Cyp1B1 in different kinds of vascular cells are cell-specific [232]. The mechanism responsible for impairment of angiogenesis relied on activation of TSP2/NF-κB and inhibition of eNOS/NO pathways, but is independent of increased oxidative stress in retinal vascular cells deficient of Cyp1B1 [233, 234]. Liver sinusoidal ECs (LSECs) play a pivotal role in endocytosis, metabolism, and scavenging circulating macromolecules from systemic circulation. Depletion of Cyp1B1 in LSECs decreased VEGF, eNOS, inflammatory mediators and adhesive ability, but increased VEGFR2 expression, cell proliferation and migration [235]. All these studies suggested that the role of Cyp1B1 in vascular function depend on cell types and the pro-angiogenic role of Cyp1B1 in ECs probably depends on VEGF/VEGFR2, eNOS pathways, and inhibition of antiangiogenic factor.

### A kinase anchor protein 1 (Akap1)

OMM protein Akap1 (also known as Akap121, dakap1) is a scaffold protein that recruits signaling proteins, such as cAMP/PKA, tyrosine phosphatase D1-Src tyrosine kinase and RNA binding proteins to the OMM, playing critical roles in maintenance of mitochondrial metabolism, protein synthesis and cell survival [236, 237]. Akap1 was widely expressed in various tissues. Down-regulation of Akap1 was identified in cardiac hypertrophy and adipose tissue from obesity associated with fatty acid β-oxidation and thermogenesis, but up-regulation in primary tumor was associated with mitochondrial metabolism and cell motility [238–241]. In HUVECs, Akap1 regulated VEGFR2 stability and inhibited its degradation through PKA/p38-dependent phosphorylation of VEGFR2 at Y1173, suggesting a fine-tuning regulatory mechanism of angiogenic signaling [242]. Akap1 knockout significantly increased ROS production and apoptosis, reduced Akt/eNOS phosphorylation, capillary-like network formation, migration and proliferation in ECs, impaired hindlimb blood flow and skeletal muscle capillary density after femoral artery ligation in mice [243], suggesting that Akap1 may serve as a novel target for improving endothelial dysfunction and treating vascular diseases.

### Ferrochelatase (FECH)

Heme acts as a signaling biomolecule and cofactor for proteins and enzymes such as cyps and iNOS that is involved in electron transport, erythropoiesis, signal transduction, biological clock, microRNA processing, and angiogenesis [244]. IMM protein ferrochelatase (FECH) inserts ferrous ion into a precursor protoporphyrin IX (PPIX) to form protoheme (iron-protoporphyrin IX) in the last step of heme biosynthesis by interaction with mitoferrin-1 and ATP-binding cassette proteins (ABCB7 and ABCB10) to form an oligomeric complex, which is regulated by the oxygen and iron status [245]. Mitochondrial superoxide reduced heme availability by disrupting iron-sulfur clusters and inhibiting FECH, which impaired cAMP/PKG and vasodilatation [246]. FECH was reported to overexpress in “wet” age-related macular degeneration eyes and murine choroidal neovascularization. FECH depletion or inhibition by griseofulvin decreased HBMECs  and HRECs proliferation, migration and tube formation, suppressed VEGFR2 through decreasing eNOS and HIF-1α expression and reduced retinal and choroidal neovascularization, but had no effect on macrovascular HUVECs proliferation, indicating the therapeutic target for choroidal neovascularization and role of FECH on angiogenesis is microvascular cell-specific [247, 248]. Furthermore, FECH inhibition decreased HRECs oxidative phosphorylation and mitochondrial complex IV, reduced mitochondrial fusion and mass, indicating for the first time that FECH-mediated mitochondrial heme metabolism and dysfunction regulated ECs angiogenesis [249]. Through high-throughput screening, a class of triazolopyrimidinone was identified to competitively inhibit FECH and angiogenesis in vitro and in vivo, indicating FECH inhibitors could be effective in treating retinal neovascularization and other diseases [250].

### Mitochondrial STAT3

STAT3 is a transcription factor that controls a hundreds of genes involved in cell proliferation, inflammation, differentiation and apoptosis in response to cytokines and growth factors. It is known that VEGF/VEGFR2 phosphorylated STAT3 at Y705, leading to the formation of heterodimers and translocation to the nucleus, and regulate ECs inflammation, permeability, extracellular matrix remodeling and intercellular communication [251–253]. Furthermore, STAT3 could translocate into the mitochondria and regulate activity of electron transport chain complexes I and II, ROS production and mPTP opening [254, 255]. Mitochondrial subcellular localization STAT3 is associated with the inner mitochondrial membrane and mitochondrial matrix. Complex I subunit gene associated with retinoid interferon induced cell mortality 19 (GRIM-19) acts as a chaperone to recruit STAT3 into mitochondria, while S727A mutation in STAT3 reduces its import in the presence of GRIM-19 [256]. ROS could act as another signaling molecule to induce p-Ser727 of STAT3 and increase its mitochondrial localization [257]. All these studies suggested that phosphorylation of STAT3 at S727 is required for its mitochondrial import. Furthermore, recent study indicated that mitochondrial ROS-nuclear STAT3-VEGF-A pathway involved in NOX2-mediated porcine vascular ECs (PVECs) tube formation and angiogenesis [258]. Indeed, mitochondrial STAT3 could interact with CypD to reduce mtROS production, whether mitochondrial STAT3 involving in EC angiogenesis and intercellular communication is unknown and requires further studies [99].

### Neuropilin 1 (NRP1)

Neuropilins (NRP 1 and NRP2) are transmembrane glycoproteins that act as co-receptors of VEGFA-165 and VEGFR, which play an important role in vascular development. NRP1 is required for VEGF-induced ECs proliferation, migration, permeability and attachment via the VEGFR2-dependent and VEGFR2-independent pathway (ECM signalling and actin remodeling) [259, 260]. Nrp1 is found primarily in arterial ECs, while Nrp2 is the venous and lymphatic endothelial marker. NRP1 is highly expressed in endothelial tip cell, and can promote tip cell rather than stalk cells during sprouting angiogenesis [261]. Furthermore, NRP1 promotes tip cels filopodia formation and actin remodeling by activation of CDC42 [262], suggesting NRP1 is responsible for cell migration rather than proliferation in VEGFR2-independent pathway. Hypoxia and nutrient deprivation decreased NRP1 expression in ECs through lysosomal degradation but did not affect NRP2 expression. In the absence of NRP1, NRP2 can function to mediate endothelial tube formation under hypoxia, suggesting NRP2 could make up the function of Nrp1 in angiogenesis [263]. Recent study showed that NRP1-deficient  human microvascular ECs (HMECs) reduced growth and increased cellular senescence. Furthermore, NRP1 was reported to locate in mitochondria and responsible for mitochondrial activity and morphology. Mitochondrial NRP1 prevented iron-dependent mitochondrial superoxide production and premature senescence through interacting with the mitochondrial transporter ATP-binding cassette B8 (ABCB8), indicating mitochondrial NRP1-ABCB8 pathway play important role in EC homeostasis by improving mitochondrial function and decreasing mtROS production [264].

### KATP channels

KATP, including pore-forming Kir6.1/6.2 subunits and regulatory sulfonylurea receptor (SUR1, SUR2A and SUR2B) subunits, are responsible for maintaining membrane potential and modulating vasoactive compounds release, vascular tone and inflammation [265]. The expression of KATP subunits such as KIR6.1 and SUR2B were decreased in hypertension and Kir6.2 polymorphism was associated with coronary microvascular dysfunction and ischemic heart disease, indicating abnormal expression of KATP channels resulted in cardiovascular-related diseases [266, 267]. KATP was reported to express in ECFCs and endothelium. Knockdown of Kir6.1 decreased HUVECs migration and network morphogenesis, while activation of KATP by nicorandil and SG-209 increased ECs proliferation and migration [268, 269]. In vivo, mice deletion of endothelial KATP subunit Kir6.1 impaired vasorelaxation during hypoxia, became more hypertensive in a high-salt diet, suggesting regulation of endothelial KATP is an important target for treatment of development of hypertension and atherosclerosis [270, 271]. In addition, although large amount of evidence about the location of KATP channels such as Kir6.1, Kir6.2, and SUR2 are in mitochondria, these are still in debate in different cells [265]. KATP channels openers 3,5,3'-Levo-triiodothyronine increased myocardial angiogenesis through mitochondrial transcription factor A, biogenesis protein peroxisome proliferator activated receptor γ coactivator-1α and activation of mitochondria KATP dependent pathway [272]. Whether mitochondria KATP involved in ECs angiogenesis and the specific mechanism need further study.

### Extracellular vesicles (EVs)

EVs are the heterogeneous plasma membrane vesicles within diameter from 30–120 nm. EVs are mediators of intercellular communication that contain multiple bioactive components such as growth factors, protein, microRNAs and nucleic acids, and activate signaling pathways in targeted cell. Exosomes derived from different cell types has been reviewed to promote or inhibit ECs angiogenesis through various mechanisms including regulating mitochondrial function in normal physiology and pathological conditions [273, 274]. For example, adipose-derived EVs promoted HUVECs tube formation and angiogenesis more than bone marrow-derived EVs, while also maximizing HUVECs mitochondrial respiration and ATP production [275]. Furthermore, the mechanism responsible for angiogenesis and wound healing from exosomes in adipose mesenchymal stem cells is dependent on SIRT3/MnSOD [276]. Exosomes contained miR-210 in EPCs also acted as a regulator of mitochondrial function to attenuate hypoxia/reoxygeneation-injured EC apoptosis, ROS overproduction and angiogenesis [277]. Moreover, EVs may directly contain mitochondria or mitochondrial compositions such as membrane protein and enzymes that regulate ECs function. EPCs may increase brain endothelial mitochondrial DNA copy number, intracellular ATP, permeability and angiogenic function through EPC-derived mitochondrial transfer after oxygen–glucose deprivation [278]. In addition, microvesicles (diameter from 100 to 1000  nm) derived from human cerebral microvascular ECs line (hCMEC/D3) increased mitochondrial functions by upregulation of OCR and ECAR under hypoxia but not EVs, suggesting microvesicles are more efficient in transferring mitochondria and regulating mitochondrial function compared with EVs [279]. Thus, mitochondria from EVs could be potentially utilized for therapeutic approaches in attenuating ECs dysfunction and promoting angiogenesis depending on its cell source and EVs characteristics.

### Sodium-glucose cotransporter 2 (SGLT2) inhibitors

SGLTs are responsible for the transport of glucose and other ions across the cell membrane. Among them, SGLT1 is responsible for 10%-20% glucose reabsorption that predominantly found in the gastrointestinal tract, while SGLT2 is primarily responsible for 80%-90% of glucose reabsorption that expressed in the proximal tubule. SGLT2 inhibitors (empagliflozin, canagliflozin and dapagliflozin) are the class of glucose-lowering drugs that have been approved to reduce cardiovascular disease and mortality [280]. The mechanisms of the cardiovascular benefits of SGLT2 inhibitors have been reviewed including improvement of endothelial function, reduction of inflammation, endoplasmic reticulum stress and oxidative stress, and regulation of metabolic shift [281]. Empagliflozin was reported to preserve cardiac microvascular ECs (CMECs) senescence and barrier function, and attenuated high glucose-induced HBMECs permeability through inhibition of mtROS, Ca^2+^ and mitochondrial fission [282, 283]. However, clinical concentrations of canagliflozin treatment inhibited HUVECs proliferation and migration by diminishing DNA synthesis and inducing cell cycle arrest, but empagliflozin or dapagliflozin did not affect cell proliferation and tube formation, explaining the side effect such as risk of limb amputations in the clinical use of this drug [284]. Whether different mechanisms of regulating mitochondrial function involved in these clinical use need further study.

## Conclusions and future perspective

The present review showed that mitochondria including proteins, metabolites and molecules act as signaling cascades and metabolic regulators of ECs function and angiogenesis, which depends on ECs subtypes, disease status, local environment and external stimulation. Furthermore, many small compounds targeting mitochondrial proteins or metabolic pathways have been designed and proved to be effective in reducing the risk and progression of endothelial dysfunction and vascular disease in vivo and in vitro. However, our understanding of the mechanism of mitochondria in regulating angiogenic signaling process is still poor. For example, the structure and biological function of some mitochondrial proteins is still unclear. Also, metabolite-driven protein post-translational modifications and environment-induced epigenetic changes have received wide attention to regulate protein function, ECs metabolism and homeostasis, their mechanisms in regulating ECs angiogenesis still need further study. In addition, what is the molecular mechanism of metabolism in different endothelial subtypes since function of some proteins are cell specific? How does these mitochondrial proteins coordinate with each other and cell–cell communications to regulate ECs metabolism and angiogenic signaling cascades in response to environmental stimulation in vivo? A better understanding of the molecular mechanisms regulating angiogenic signaling will generate a new perspective in disease pathogenesis and treatment. Furthermore, design and application of specific mitochondria-targeted compounds to regulate ECs angiogenesis and function in vivo and clinical trials are urgent and hold promise for treatment of angiogenesis-related diseases and preserving vascular health.

## Data Availability

All data generated during this study are included in this article.

## References

[1] Herbert SP, Stainier DYR (2011). Molecular control of endothelial cell behaviour during blood vessel morphogenesis. Nat Rev Mol Cell Biol.

[2] Reynolds LP, Grazul-Bilska AT, Redmer DA (2002). Angiogenesis in the female reproductive organs: pathological implications. Int J Exp Pathol.

[3] Dromparis P, Michelakis ED (2013). Mitochondria in vascular health and disease. Annu Rev Physiol.

[4] De Smet F, Segura I, De Bock K (2009). Mechanisms of vessel branching. Arterioscler Thromb Vasc Biol.

[5] Potente M, Gerhardt H, Carmeliet P (2011). Basic and therapeutic aspects of angiogenesis. Cell.

[6] Potente M, Carmeliet P (2017). The link between angiogenesis and endothelial metabolism. Annu Rev Physiol.

[7] Huang Z, Huang S, Song T, Yin Y, Tan C (2021). Placental angiogenesis in mammals: a review of the regulatory effects of signaling pathways and functional nutrients. Adv Nutr.

[8] Culic O, Gruwel ML, Schrader J (1997). Energy turnover of vascular endothelial cells. Am J Physiol Cell Physiol.

[9] Nomura M, Yamagishi SI, Harada SI, Hayashi Y, Yamashima T, Yamashita J, Yamamoto H (1995). Possible participation of autocrine and paracrine vascular endothelial growth factors in hypoxia-induced proliferation of endothelial cells and pericytes. J Biol Chem.

[10] Caja S, Enríquez JA (2017). Mitochondria in endothelial cells: sensors and integrators of environmental cues. Redox Biol.

[11] Sun D, Wang J, Toan S, Muid D, Li R, Chang X, Zhou H (2022). Molecular mechanisms of coronary microvascular endothelial dysfunction in diabetes mellitus: focus on mitochondrial quality surveillance. Angiogenesis.

[12] Kluge MA, Fetterman JL, Vita JA (2013). Mitochondria and endothelial function. Circ Res.

[13] Panieri E, Santoro MM (2015). ROS signaling and redox biology in endothelial cells. Cell Mol Life Sci.

[14] Connor KM, Subbaram S, Regan KJ, Nelson KK, Mazurkiewicz JE, Bartholomew PJ, Aplin AE, Tai Y-T, Aguirre-Ghiso J, Flores SC, Melendez JA (2005). Mitochondrial H2O2 regulates the angiogenic phenotype via PTEN oxidation. J Biol Chem.

[15] Marrotte EJ, Chen D-D, Hakim JS, Chen AF (2010). Manganese superoxide dismutase expression in endothelial progenitor cells accelerates wound healing in diabetic mice. J Clin Invest.

[16] Teixeira RB, Pfeiffer M, Zhang P, Shafique E, Rayta B, Karbasiafshar C, Ahsan N, SellkeAbid FWMR (2023). Reduction in mitochondrial ROS improves oxidative phosphorylation and provides resilience to coronary endothelium in non-reperfused myocardial infarction. Basic Res Cardiol.

[17] Ungvari Z, Labinskyy N, Mukhopadhyay P, Pinto JT, Bagi Z, Ballabh P, Zhang C, Pacher P, Csiszar A (2009). Resveratrol attenuates mitochondrial oxidative stress in coronary arterial endothelial cells. Am J Physiol Heart C.

[18] Agostini S, Chiavacci E, Matteucci M, Torelli M, Pitto L, Lionetti V (2015). Barley beta-glucan promotes MnSOD expression and enhances angiogenesis under oxidative microenvironment. J Cell Mol Med.

[19] Wang Y, Zang QS, Liu Z (2011). Regulation of VEGF-induced endothelial cell migration by mitochondrial reactive oxygen species. Am J Physiol Cell Physiol.

[20] Wang H, Hartnett ME (2017). Roles of nicotinamide adenine dinucleotide phosphate (NADPH) oxidase in angiogenesis: isoform-specific effects. Antioxidants.

[21] Fukai T, Ushio-Fukai M (2020). Cross-talk between NADPH oxidase and mitochondria: role in ROS signaling and angiogenesis. Cells.

[22] Evangelista AM, Thompson MD, Bolotina VM, Tong X, Cohen RA (2012). Nox4- and Nox2-dependent oxidant production is required for VEGF-induced SERCA cysteine-674 S-glutathiolation and endothelial cell migration. Free Radic Bio Med.

[23] Kim Y-M, Kim S-J, Tatsunami R, Yamamura H, Fukai T, Ushio-Fukai M (2017). ROS-induced ROS release orchestrated by Nox4, Nox2, and mitochondria in VEGF signaling and angiogenesis. Am J Physiol Cell Physiol.

[24] Shafique E, Torina A, Reichert K, Colantuono B, Nur N, Zeeshan K, Ravichandran V, Liu Y, Feng J, Zeeshan K, Benjamin LE, Irani K, Harrington EO, Sellke FW, Abid MR (2017). Mitochondrial redox plays a critical role in the paradoxical effects of NAPDH oxidase-derived ROS on coronary endothelium. Cardiovasc Res.

[25] Semenza GL (2012). Hypoxia-inducible factors in physiology and medicine. Cell.

[26] Orr AL, Vargas L, Turk CN, Baaten JE, Matzen JT, Dardov VJ, Attle SJ, Li J, Quackenbush DC, Goncalves RLS, Perevoshchikova IV, Petrassi HM, Meeusen SL, Ainscow EK, Brand MD (2015). Suppressors of superoxide production from mitochondrial complex III. Nat Chem Biol.

[27] Bell EL, Klimova TA, Eisenbart J, Moraes CT, Murphy MP, Budinger GRS, Chandel NS (2007). The Qo site of the mitochondrial complex III is required for the transduction of hypoxic signaling via reactive oxygen species production. J Cell Biol.

[28] Lin X, David CA, Donnelly JB, Michaelides M, Chandel NS, Huang X, Warrior U, Weinberg F, Tormos KV, Fesik SW, Shen Y (2008). A chemical genomics screen highlights the essential role of mitochondria in HIF-1 regulation. Proc Natl Acad Sci USA.

[29] Pearlstein DP, Ali MH, Mungai PT, Hynes KL, Gewertz BL, Schumacker PT (2002). Role of mitochondrial oxidant generation in endothelial cell responses to hypoxia. Arterioscler Thromb Vasc Biol.

[30] Hernansanz-Agustín P, Ramos E, Navarro E, Parada E, Sánchez-López N, Peláez-Aguado L, Cabrera-García JD, Tello D, Buendia I, Marina A, Egea J, López MG, Bogdanova A, Martínez-Ruiz A (2017). Mitochondrial complex I deactivation is related to superoxide production in acute hypoxia. Redox Biol.

[31] Hernansanz-Agustín P, Izquierdo-Álvarez A, Sánchez-Gómez FJ, Ramos E, Villa-Piña T, Lamas S, Bogdanova A, Martínez-Ruiz A (2014). Acute hypoxia produces a superoxide burst in cells. Free Radic Biol Med..

[32] Hernansanz-Agustín P, Choya-Foces C, Carregal-Romero S (2020). Na+ controls hypoxic signalling by the mitochondrial respiratory chain. Nature.

[33] Lee G, Won HS, Lee YM, Choi JW, Oh TI, Jang JH, Choi DK, Lim B-O, Kim YJ, Park J-W, Puigserver P, Lim JH (2016). Oxidative dimerization of PHD2 is responsible for its inactivation and contributes to metabolic reprogramming via HIF-1α activation. Sci Rep.

[34] Masson N, Singleton RS, Sekirnik R, Trudgian DC, Ambrose LJ, Miranda MX, Tian YM, Kessler BM, Schofield CJ, Ratcliffe PJ (2012). The FIH hydroxylase is a cellular peroxide sensor that modulates HIF transcriptional activity. EMBO Rep.

[35] Napoli C, Martin-Padura I, de Nigris F, Giorgio M, Mansueto G, Somma P, Condorelli M, Sica G, Rosa GD, Pelicci P (2003). Deletion of the p66Shc longevity gene reduces systemic and tissue oxidative stress, vascular cell apoptosis, and early atherogenesis in mice fed a high-fat diet. Proc Natl Acad Sci USA.

[36] Camici GG, Schiavoni M, Francia P, Cosentino F (2007). Genetic deletion of p66Shc adaptor protein prevents hyperglycemia-induced endothelial dysfunction and oxidative stress. Proc Natl Acad Sci USA.

[37] Oshikawa J, Kim S-J, Furuta E, Caliceti C, Chen G-F, McKinney RD, Kuhr F, Levitan I, Fukai T, Ushio-Fukai M (2011). Novel role of p66Shc in ROS-dependent VEGF signaling and angiogenesis in endothelial cells. Am J Physiol Heart Circ Physiol.

[38] Mishra M, Duraisamy AJ, Bhattacharjee S, Kowluru RA (2018). Adaptor protein p66Shc: a link between cytosolic and mitochondrial dysfunction in the development of diabetic retinopathy. Antioxid Redox Signal.

[39] Yamamori T, White AR, Mattagajasingh I, Khanday FA, Haile A, Qi B, Jeon BH, Bugayenko A, Kasuno K, Berkowitz DE, Irani K (2005). P66shc regulates endothelial NO production and endothelium-dependent vasorelaxation: implications for age-associated vascular dysfunction. J Mol Cell Cardiol.

[40] Shi Y, Cosentino F, Camici GG, Akhmedov A, Vanhoutte PM, Tanner FC, Lüscher TF (2011). Oxidized low-density lipoprotein activates p66Shc via lectin-like oxidized low-density lipoprotein receptor-1, protein kinase C-β, and c-Jun N-terminal kinase kinase in human endothelial cells. Arterioscler Thromb Vasc Biol.

[41] Paneni F, Mocharla P, Akhmedov A, Costantino S, Osto E, Volpe M, Lüscher TF, Cosentino F (2012). Gene silencing of the mitochondrial adaptor p66Shc suppresses vascular hyperglycemic memory in diabetes. Circ Res.

[42] Shi Y, Luescher TF, Camici GG (2014). Dual role of endothelial nitric oxide synthase in oxidized LDL-induced, p66(Shc)-mediated oxidative stress in cultured human endothelial cells. PLoS ONE.

[43] Kim C-S, Jung S-B, Naqvi A, Hoffman TA, DeRicco J, Yamamori T, Cole MP, Jeon B-H, Irani K (2008). P53 impairs endothelium-dependent vasomotor function through transcriptional upregulation of p66shc. Circ Res.

[44] Kumar S, Kim Y-R, Vikram A, Naqvi A, Li Q, Kassan M, Kumar V, Bachschmid MM, JacobsJS KA, Irani K (2017). Sirtuin1-regulated lysine acetylation of p66Shc governs diabetes-induced vascular oxidative stress and endothelial dysfunction. Proc Natl Acad Sci USA.

[45] Kim Y-R, Kim C-S, Naqvi A, Kumar A, Kumar S, Hoffman TA, Irani K (2012). Epigenetic upregulation of p66shc mediates low-density lipoprotein cholesterol-induced endothelial cell dysfunction. Am J Physiol Heart Circ Physiol.

[46] Haslem L, Hays JM, Hays FA (2022). p66Shc in cardiovascular pathology. Cells..

[47] Jung HJ, Shim JS, Lee J, Song YM, Park KC, Choi SH, Kim ND, Yoon JH, Mungai PT, Schumacker PT, Kwon HJ (2010). Terpestacin inhibits tumor angiogenesis by targeting UQCRB of mitochondrial complex III and suppressing hypoxia-induced reactive oxygen species production and cellular oxygen sensing. J Biol Chem.

[48] Cho YS, Jung HJ, Seok SH, Payumo AY, Chen JK, Kwon HJ (2013). Functional inhibition of UQCRB suppresses angiogenesis in zebrafish. Biochem Biophys Res Commun.

[49] Jung HJ, Kim Y, Chang J, Kang SW, Kim JH, Kwon HJ (2013). Mitochondrial UQCRB regulates VEGFR2 signaling in endothelial cells. J Mol Med.

[50] Diebold LP, Gil HJ, Gao P, Martinez CA, Weinberg SE, Chandel NS (2019). Mitochondrial complex III is necessary for endothelial cell proliferation during angiogenesis. Nat Metab.

[51] Sun YH, Zhang XY, Xie WQ, Liu GJ, He XX, Huang YL, Zhang GX, Wang J, Kuang ZY, Zhang R (2016). Identification of UQCRB as an oxymatrine recognizing protein using a T7 phage display screen. J Ethnopharmacol..

[52] Jung HJ, Kim KH, Kim ND, Han G, Kwon HJ (2011). Identification of a novel small molecule targeting UQCRB of mitochondrial complex III and its anti-angiogenic activity. Bioorg Med Chem Lett.

[53] Guo Y-J, Chen L, Bai Y-P, Li L, Sun J, Zhang G-G, Yang T-L, Xia J, Li Y-J, Chen X-P (2010). The ALDH2 Glu504Lys polymorphism is associated with coronary artery disease in Han Chinese: relation with endothelial ADMA levels. Atherosclerosis.

[54] Liu X, Sun X, Liao H, Dong Z, Zhao J, Zhu H, Wang P, Shen L, Xu L, Ma X, Shen C, Fan F, Wang C, Hu K, Zou Y, Ge J, Ren J, Sun A (2015). Mitochondrial aldehyde dehydrogenase 2 regulates revascularization in chronic ischemia. Arterioscler Thromb Vasc Biol.

[55] Pan G, Roy B, Palaniyandi SS (2021). Diabetic aldehyde dehydrogenase 2 mutant (ALDH2*2) mice are more susceptible to cardiac ischemic-reperfusion injury due to 4-Hydroxy-2-nonenal induced coronary endothelial cell damage. J Am Heart Assoc.

[56] Kimura M, Yokoyama A, Higuchi S (2019). Aldehyde dehydrogenase-2 as a therapeutic target. Expert Opin Ther Targets.

[57] Li S-Y, Gomelsky M, Duan J, Zhang Z, Gomelsky L, Zhang X, Epstein PN, Ren J (2004). Overexpression of aldehyde dehydrogenase-2 (ALDH2) transgene prevents acetaldehyde-induced cell injury in human umbilical vein endothelial cells: role of ERK AND p38 mitogen-activated protein kinase. J Biol Chem.

[58] Solito R, Corti F, Chen C-H, Mochly-Rosen D, Giachetti A, Ziche M (2013). Donnini S (2013) Mitochondrial aldehyde dehydrogenase-2 activation prevents β-amyloid-induced endothelial cell dysfunction and restores angiogenesis. J Cell Sci.

[59] Xue L, Xu F, Meng L, Wei S, Wang J, Hao P, Bian Y, Zhang Y, Chen Y (2012). Acetylation-dependent regulation of mitochondrial ALDH2 activation by SIRT3 mediates acute ethanol-induced eNOS activation. FEBS Lett.

[60] Capoccia BJ, Robson DL, Levac KD, Maxwell DJ, Hohm SA, Neelamkavil MJ, Bell GI, Xenocostas A, Link DC, Piwnica-Worms D, Nolta JA, Hess DA (2009). Revascularization of ischemic limbs after transplantation of human bone marrow cells with high aldehyde dehydrogenase activity. Blood.

[61] Povsic TJ, Zavodni KL, Kelly FL, Zhu S, Goldschmidt-Clermont PJ, Dong C, Peterson ED (2007). Circulating progenitor cells can be reliably identified on the basis of aldehyde dehydrogenase activity. J Am Coll Cardiol.

[62] Zhao Y, Wang B, Zhang J, He D, Zhang Q, Pan C, Yuan Q, Shi Y, Tang H, Xu F, Wei S, Chen Y (2019). ALDH2 (Aldehyde dehydrogenase 2) protects against hypoxia-induced pulmonary hypertension. Arterioscler Thromb Vasc Biol.

[63] Dunn LL, Buckle AM, Cooke JP, Ng MKC (2010). The emerging role of the thioredoxin system in angiogenesis. Arterioscler Thromb Vasc Biol.

[64] Zhou J, Damdimopoulos AE, Spyrou G, Brüne B (2007). Thioredoxin 1 and thioredoxin 2 have opposed regulatory functions on hypoxia-inducible factor-1α. J Biol Chem.

[65] Zhang H, Luo Y, Zhang W, Dai S, Zhang R, Huang Y, Bernatchez P, Giordano FJ, Shadel G, Sessa WC, Min W (2007). Endothelial-specific expression of mitochondrial thioredoxin improves endothelial cell function and reduces atherosclerotic lesions. Am J Pathol.

[66] Dai S, He Y, Zhang H, Yu L, Wan T, Xu Z, Jones D, Chen H, Min W (2009). Endothelial-specific expression of mitochondrial thioredoxin promotes ischemia-mediated arteriogenesis and angiogenesis. Arterioscler Thromb Vasc Biol.

[67] Liu Y, Min W (2002). Thioredoxin promotes ASK1 ubiquitination and degradation to inhibit ASK1-mediated apoptosis in a redox activity-independent manner. Circ Res.

[68] Zhang R, Al-Lamki R, Bai L, Streb JW, Miano JM, Bradley J, Min W (2004). Thioredoxin-2 inhibits mitochondria-located ASK1-mediated apoptosis in a JNK-independent manner. Circ Res.

[69] Kirsch J, Schneider H, Pagel J-I (2016). Endothelial dysfunction, and a prothrombotic, proinflammatory phenotype is caused by loss of mitochondrial thioredoxin reductase in endothelium. Arterioscler Thromb Vasc Biol.

[70] Chasapis CT, Makridakis M, Damdimopoulos AE, Zoidakis J, Lygirou V, Mavroidis M, Vlahou A, Miranda-Vizuete A, Spyrou G, Vlamis-Gardikas A (2019). Implications of the mitochondrial interactome of mammalian thioredoxin 2 for normal cellular function and disease. Free Radic Biol Med.

[71] Pirozzi CJ, Yan H (2021). The implications of IDH mutations for cancer development and therapy. Nat Rev Clin Oncol.

[72] Wang X, Chen Z, Xu J (2022). SLC1A1-mediated cellular and mitochondrial influx of R-2-hydroxyglutarate in vascular endothelial cells promotes tumor angiogenesis in IDH1-mutant solid tumors. Cell Res.

[73] Kim SH, Park JW (1865). IDH2 deficiency impairs cutaneous wound healing via ROS-dependent apoptosis. Biochim Biophys Acta Mol Basis Dis.

[74] Park JB, Nagar H, Choi S, Jung SB, Kim HW, Kang SK, Lee JW, Lee JH, Park JW, Irani K, Jeon BH, Song HJ, Kim CS (2016). IDH2 deficiency impairs mitochondrial function in endothelial cells and endothelium-dependent vasomotor function. Free Radic Biol Med.

[75] Choi SJ, Piao S, Nagar H, Jung SB, Kim S, Lee I, Kim SM, Song HJ, Shin N, Kim DW, Irani K, Jeon BH, Park JW, Kim CS (2018). Isocitrate dehydrogenase 2 deficiency induces endothelial inflammation via p66sh-mediated mitochondrial oxidative stress. Biochem Biophys Res Commun.

[76] Kim SH, Kim H, Ku HJ, Park JH, Cha H, Lee S, Lee JH, Park J-W (2016). Oxalomalate reduces expression and secretion of vascular endothelial growth factor in the retinal pigment epithelium and inhibits angiogenesis: implications for age-related macular degeneration. Redox Biol.

[77] Yokota Y, Nakajima H, Wakayama Y, Muto A, Kawakami K, Fukuhara S, Mochizuki N (2015). Endothelial Ca2+ oscillations reflect VEGFR signaling-regulated angiogenic capacity in vivo. Elife.

[78] Noren DP, Chou WH, Lee SH, Qutub AA, Warmflash A, Wagne DS, Popel AS, Levchenko A (2016). Endothelial cells decode VEGF-mediated Ca2+ signaling patterns to produce distinct functional responses. Sci Signal..

[79] Li J, Cubbon RM, Wilson LA, Amer MS, McKeown L, Hou B, Majeed Y, Tumova S, Seymour VAL, Taylor H, Stacey M, O'Regan D, Foster R, Porter KE, Kearney MT, Beech DJ (2011). Orai1 and CRAC channel dependence of VEGF-activated Ca2+ entry and endothelial tube formation. Circ Res.

[80] Favia A, Desideri M, Gambara G, D'Alessio A, Ruas M, Esposito B, Bufalo DD, Parrington J, Ziparo E, Palombi F, Galione A, Filippini A (2014). VEGF-induced neoangiogenesis is mediated by NAADP and two-pore channel-2–dependent Ca2+ signaling. Proc Natl Acad Sci USA.

[81] Mittal M, Urao N, Hecquet CM, Zhang M, Sudhahar V, Gao X, Komarova Y, Ushio-Fukai M, Malik AB (2015). Novel role of reactive oxygen species–activated trp melastatin channel-2 in mediating angiogenesis and postischemic neovascularization. Arterioscler Thromb Vasc Biol.

[82] Rizzuto R, De Stefani D, Raffaello A, Mammucari C (2012). Mitochondria as sensors and regulators of calcium signalling. Nat Rev Mol Cell Biol.

[83] Wang Y, Huang Y, Liu Y, Li J, Hao Y, Yin P, Liu Z, Chen J, Wang Y, Wang N, Zhang P (2018). Microtubule associated tumor suppressor 1 interacts with mitofusins to regulate mitochondrial morphology in endothelial cells. FASEB J.

[84] Zheng X, Lu S, He Z, Huang H, Yao Z, Miao Y, Cai C, Fei Z (2020). MCU-dependent negative sorting of miR-4488 to extracellular vesicles enhances angiogenesis and promotes breast cancer metastatic colonization. Oncogene.

[85] Dedkova EN, Ji X, Lipsius SL, Blatter LA (2004). Mitochondrial calcium uptake stimulates nitric oxide production in mitochondria of bovine vascular endothelial cells. Am J Physiol-Cell Physiol.

[86] Zinghirino F, Pappalardo XG, Messina A, Nicosia G, Pinto VD, Guarino F (2021). VDAC genes expression and regulation in mammals. Front Physiol.

[87] Maldonado EN (2017). VDAC–tubulin, an anti-Warburg pro-oxidant switch. Front Oncol.

[88] Geisler S, Holmström KM, Skujat D, Fiesel FC, Rothfuss OC, Kahle PJ, Springer W (2010). PINK1/Parkin-mediated mitophagy is dependent on VDAC1 and p62/SQSTM1. Nat Cell Biol.

[89] Dadsena S, Bockelmann S, Mina JGM, Hassan DG, Korneev S, Razzera G, Jahn H, Niekamp P, Müller D, Schneider M, Tafesse FG, Marrink SJ, Melo MN, Holthuis JCM (2019). Ceramides bind VDAC2 to trigger mitochondrial apoptosis. Nat Commun.

[90] Tomasello F, Messina A, Lartigue L, Schembri L, Medina C, Reina S, Thoraval D, Crouzet M, Ichas F, Pinto VD, Giorgi FD (2009). Outer membrane VDAC1 controls permeability transition of the inner mitochondrial membrane in cellulo during stress-induced apoptosis. Cell Res.

[91] Alvira CM, Umesh A, Husted C, Ying L, Hou Y, Lyu S-C, Nowak J, Cornfield DN (2012). Voltage-dependent anion channel-2 interaction with nitric oxide synthase enhances pulmonary artery endothelial cell nitric oxide production. Am J Respir Cell Mol Biol.

[92] Head Sarah A, Shi W, Zhao L, Gorshkov K, Pasunooti K, Chen Y, Deng Z, Li R, Shim JS, Tan W, Hartung T, Zhang J, Zhao Y, Colombini M, Liu JO (2015). Antifungal drug itraconazole targets VDAC1 to modulate the AMPK/mTOR signaling axis in endothelial cells. Proc Natl Acad Sci USA.

[93] Carreira RS, Lee Y, Ghochani M, Gustafsson AB, Gottlieb RA (2010). Cyclophilin D is required for mitochondrial removal by autophagy in cardiac cells. Autophagy.

[94] Nakagawa T, Shimizu S, Watanabe T, Yamaguchi O, Otsu K, Yamagata H, Inohara H, Kubo T, Tsujimoto Y (2005). Cyclophilin D-dependent mitochondrial permeability transition regulates some necrotic but not apoptotic cell death. Nature.

[95] Shanmughapriya S, Rajan S, Hoffman Nicholas E (2015). SPG7 Is an essential and conserved component of the mitochondrial permeability transition pore. Mol Cell.

[96] Guo L (2022). Mitochondrial ATP synthase inhibitory factor 1 interacts with the p53–cyclophilin D complex and promotes opening of the permeability transition pore. J Biol Chem.

[97] Leung AWC, Varanyuwatana P, Halestrap AP (2008). The Mitochondrial phosphate carrier interacts with cyclophilin D and may play a key role in the permeability transition. J Biol Chem.

[98] Tavecchio M, Lisanti S, Lam A, Ghosh JC, Martin NM, O'Connell M, Weeraratna AT, Kossenkov AV, Showe LC, Altieri DC (2013). Cyclophilin D extramitochondrial eignaling controls cell cycle progression and chemokine-directed cell motility. J Biol Chem.

[99] Meier JA, Hyun M, Cantwell M, Raza A, Mertens C, Raje V, Sisler J, Tracy E, Torres-Odio S, Gispert S, Shaw PE, Baumann H, Bandyopadhyay D, Takabe K, Larner AC (2017). Stress-induced dynamic regulation of mitochondrial STAT3 and its association with cyclophilin D reduce mitochondrial ROS production. Sci Signal.

[100] Marcu R, Kotha S, Zhi Z, Qin W, Neeley CK, Wang RK, Zheng Y, Hawkins BJ (2015). The mitochondrial permeability transition pore regulates endothelial bioenergetics and angiogenesis. Circ Res.

[101] Itani HA, Dikalova AE, McMaster WG, Nazarewicz RR, Bikineyeva AT, Harrison DG, Dikalov SI (2016). Mitochondrial cyclophilin D in vascular oxidative stress and hypertension. Hypertension.

[102] Nguyen TM, Wong R, Menazza S, Sun J, Chen Y, Wang G, Gucek M, Steenbergen C, Sack MN, Murphy E (2013). Cyclophilin D modulates mitochondrial acetylome. Circ Res.

[103] Hurst S, Gonnot F, Dia M, Crola Da Silva C, Gomez L, Sheu S-S (2020). Phosphorylation of cyclophilin D at serine 191 regulates mitochondrial permeability transition pore opening and cell death after ischemia-reperfusion. Cell Death Dis.

[104] Parks RJ, Menazza S, Holmström KM, Amanakis G, Fergusson M, Ma H, Aponte AM, Bernardi P, Finkel T, Murphy E (2019). Cyclophilin D-mediated regulation of the permeability transition pore is altered in mice lacking the mitochondrial calcium uniporter. Cardiovasc Res.

[105] Bochaton T, Crola-Da-Silva C, Pillot B, Villedieu C, Ferreras L, Alam MR, Thibault H, Strina M, Gharib A, Ovize M, Baetz D (2015). Inhibition of myocardial reperfusion injury by ischemic postconditioning requires sirtuin 3-mediated deacetylation of cyclophilin D. J Mol Cell Cardiol..

[106] Bibli S-I, Papapetropoulos A, Iliodromitis EK, Daiber A, Randriamboavonjy V, Steven S, Brouckaert P, Chatzianastasiou A, Kypreos KE, Hausenloy DJ, Fleming I, Andreadou I (2019). Nitroglycerine limits infarct size through S-nitrosation of cyclophilin D: a novel mechanism for an old drug. Cardiovasc Res.

[107] Amanakis G, Sun J, Fergusson MM, McGinty S, Liu C, Molkentin JD, Murphy E (2021). Cysteine 202 of cyclophilin D is a site of multiple post-translational modifications and plays a role in cardioprotection. Cardiovasc Res.

[108] Gatliff J, Campanella M (2016). TSPO: kaleidoscopic 18-kDa amid biochemical pharmacology, control and targeting of mitochondria. Biochem J.

[109] Li Y, Chen L, Li L, Sottas C, Petrillo SK, Lazaris A, Metrakos P, Wu H, Ishida Y, Saito T, Golden-Mason L, Rosen HR, Wolff JJ, Silvescu CI, Garza S, Cheung G, Huang T, Fan J, Culty M, Stiles B, Asahina K, Papadopoulos V (2021). Cholesterol-binding translocator protein TSPO regulates steatosis and bile acid synthesis in nonalcoholic fatty liver disease. iScience.

[110] Liu J, Rone MB, Papadopoulos V (2006). Protein-protein interactions mediate mitochondrial cholesterol transport and steroid biosynthesis. J Biol Chem.

[111] Fan J, Campioli E, Midzak A, Culty M, Papadopoulos V (2015). Conditional steroidogenic cell-targeted deletion of TSPO unveils a crucial role in viability and hormone-dependent steroid formation. Proc Natl Acad Sci USA.

[112] Jaipuria G, Leonov A, Giller K, Vasa SK, Jaremko Ł, Jaremko M, Linser R, Becker S, Zweckstetter M (2017). Cholesterol-mediated allosteric regulation of the mitochondrial translocator protein structure. Nat Commun.

[113] Selvaraj V, Stocco DM (2015). The changing landscape in translocator protein (TSPO) function. Trends Endocrinol Metab.

[114] Gatliff J, East DA, Singh A, Alvarez MS, Frison M, Matic I, Ferraina C, Natalie S, Turkheimer F, Campanella M (2017). A role for TSPO in mitochondrial Ca2+ homeostasis and redox stress signaling. Cell Death Dis.

[115] Desai R, East DA, Hardy L (2020). Mitochondria form contact sites with the nucleus to couple prosurvival retrograde response. Sci Adv.

[116] Wolf A, Herb M, Schramm M, Langmann T (2020). The TSPO-NOX1 axis controls phagocyte-triggered pathological angiogenesis in the eye. Nat Commun.

[117] Fu Y, Wang D, Wang H, Cai M, Li C, Zhang X, Chen H, Hu Y, Zhang X, Ying M, He W, Zhang J (2020). TSPO deficiency induces mitochondrial dysfunction, leading to hypoxia, angiogenesis, and a growth-promoting metabolic shift toward glycolysis in glioblastoma. Neuro-Oncol.

[118] Li J, Zhang Z, Lv L, Qiao H, Chen X, Zou C (2016). A bispecific antibody (ScBsAbAgn-2/TSPO) target for Ang-2 and TSPO resulted in therapeutic effects against glioblastomas. Biochem Biophys Res Commun.

[119] Wang C, Chi Y, Li J (2014). FAM3A activates PI3K p110α/Akt signaling to ameliorate hepatic gluconeogenesis and lipogenesis. Hepatology.

[120] Xu W, Liang M, Zhang Y, Huang K, Wang C (2019). Endothelial FAM3A positively regulates post-ischaemic angiogenesis. EBioMedicine.

[121] Katsouda A, Bibli S-I, Pyriochou A, Szabo C, Papapetropoulos A (2016). Regulation and role of endogenously produced hydrogen sulfide in angiogenesis. Pharmacol Res.

[122] Polhemus DJ, Lefer DJ (2014). Emergence of hydrogen sulfide as an endogenous gaseous signaling molecule in cardiovascular disease. Circ Res.

[123] Coletta C, Papapetropoulos A, Erdelyi K, Olah G, Módis K, Panopoulos P, Asimakopoulou A, Gerö D, Sharina I, Martin E, Szabo C (2012). Hydrogen sulfide and nitric oxide are mutually dependent in the regulation of angiogenesis and endothelium-dependent vasorelaxation. Proc Natl Acad Sci USA.

[124] Weber GJ, Pushpakumar S, Tyagi SC, Sen U (2016). Homocysteine and hydrogen sulfide in epigenetic, metabolic and microbiota related renovascular hypertension. Pharmacol Res.

[125] Fu M, Zhang W, Wu L, Yang G, Li H, Wang R (2012). Hydrogen sulfide (H2S) metabolism in mitochondria and its regulatory role in energy production. Proc Natl Acad Sci USA.

[126] Panagaki T, Randi EB, Augsburger F, Szabo C (2019). Overproduction of H2S, generated by CBS, inhibits mitochondrial Complex IV and suppresses oxidative phosphorylation in Down syndrome. Proc Natl Acad Sci USA.

[127] Abdollahi Govar A, Törő G, Szaniszlo P, Pavlidou A, Bibli S-I, Thanki K, Resto VA, Chao C, Hellmich MR, Szabo C, Papapetropoulos A, Módis K (2020). 3-Mercaptopyruvate sulfurtransferase supports endothelial cell angiogenesis and bioenergetics. Br Jpharmacol.

[128] Suzuki K, Olah G, Modis K, Coletta C, Kulp G, Gerö D, Szoleczky P, Chang T, Zhou Z, Wu L, Wang R, Papapetropoulos A, Szabo C (2011). Hydrogen sulfide replacement therapy protects the vascular endothelium in hyperglycemia by preserving mitochondrial function. Proc Natl Acad Sci USA.

[129] King AL, Polhemus DJ, Bhushan S (2014). Hydrogen sulfide cytoprotective signaling is endothelial nitric oxide synthase-nitric oxide dependent. Proc Natl Acad Sci USA.

[130] Saha S, Chakraborty PK, Xiong X, Dhar Dwivedi SK, Mustafi SB, Leigh NR, Ramchandran R, Mukherjee P, Bhattacharya R (2016). Cystathionine β-synthase regulates endothelial function via protein S-sulfhydration. FASEB J.

[131] Covarrubias AE, Lecarpentier E, Lo A, Salahuddin S, Gray KJ, Karumanchi SA, Zsengellér ZK (2019). AP39, a modulator of mitochondrial bioenergetics, reduces antiangiogenic response and oxidative stress in hypoxia-exposed trophoblasts: relevance for preeclampsia pathogenesis. Am J Pathol.

[132] Gerő D, Torregrossa R, Perry A, Waters A, Le-Trionnaire S, Whatmore JL, Wood M, Whiteman M (2016). The novel mitochondria-targeted hydrogen sulfide (H2S) donors AP123 and AP39 protect against hyperglycemic injury in microvascular endothelial cells in vitro. Pharmacol Res.

[133] Módis K, Coletta C, Erdélyi K, Papapetropoulos A, Szabo C (2013). Intramitochondrial hydrogen sulfide production by 3-mercaptopyruvate sulfurtransferase maintains mitochondrial electron flow and supports cellular bioenergetics. FASEB J.

[134] Kumar R, Landry AP, Guha A, Vitvitsky V, Lee HJ, Seike K, Reddy P, Lyssiotis CA, Banerjee R (2022). A redox cycle with complex II prioritizes sulfide quinone oxidoreductase-dependent H2S oxidation. J Biol Chem.

[135] Paul BD, Snyder SH, Kashfi K (2021). Effects of hydrogen sulfide on mitochondrial function and cellular bioenergetics. Redox Biol.

[136] Sprott D, Poitz DM, Korovina I, Ziogas A, Phieler J, Chatzigeorgiou A, Mitroulis I, Deussen A, Chavakis T, Ameln AK (2019). Endothelial-specific deficiency of ATG5 (autophagy protein 5) attenuates ischemia-related angiogenesis. Arterioscler Thromb Vasc Biol.

[137] Du J, Teng RJ, Guan T, Eis A, Kaul S, Konduri GG, Shi Y (2012). Role of autophagy in angiogenesis in aortic endothelial cells. Am J Physiol-Cell Ph.

[138] Lee B, Shin H, Oh J-E, Park J, Park M, Yang SC, Jun J-H, Hong S-H, Song H, Lim HJ (2021). An autophagic deficit in the uterine vessel microenvironment provokes hyperpermeability through deregulated VEGFA, NOS1, and CTNNB1. Autophagy.

[139] Schaaf MB, Houbaert D, Meçe O, Agostinis P (2019). Autophagy in endothelial cells and tumor angiogenesis. Cell Death Differ.

[140] Ashrafi G, Schwarz TL (2013). The pathways of mitophagy for quality control and clearance of mitochondria. Cell Death Differ.

[141] Eiyama A, Okamoto K (2015). PINK1/Parkin-mediated mitophagy in mammalian cells. Curr Opin Cell Biol.

[142] Murata H, Sakaguchi M, Jin Y, Sakaguchi Y, Futami J, Yamada H, Kataoka K, Huh N (2011). A new cytosolic pathway from a Parkinson disease-associated kinase, BRPK/PINK1 ACTIVATION OF AKT VIA MTORC2. J Biol Chem.

[143] Nakajima A, Kataoka K, Hong M, Sakaguchi M, Huh N (2003). BRPK, a novel protein kinase showing increased expression in mouse cancer cell lines with higher metastatic potential. Cancer Lett.

[144] Billia F, Hauck L, Konecny F, Rao V, Shen J, Mak TW (2011). PTEN-inducible kinase 1 (PINK1)/Park6 is indispensable for normal heart function. Proc Natl Acad Sci USA.

[145] Liu J, Zhang C, Zhao Y, Yue X, Wu H, Huang S, Chen J, Tomsky K, Xie H, Khella CA, Gatza ML, Xia D, Gao J, White E, Haffty BG, Hu W, Feng Z (2017). Parkin targets HIF-1α for ubiquitination and degradation to inhibit breast tumor progression. Nat Commun..

[146] Lei Z, Duan H, Zhao T, Zhang Y, Li G, Meng J, Zhang S, Yan W (2018). PARK2 inhibits osteosarcoma cell growth through the JAK2/STAT3/VEGF signaling pathway. Cell Death Dis.

[147] Xia W, Yin J, Zhang S, Guo C, Li Y, Zhang Y, Zhang A, Jia Z, Chen H (2018). Parkin modulates ERR alpha/eNOS signaling pathway in endothelial cells. Cell Physiol Biochem.

[148] Liu L, Feng D, Chen G (2012). Mitochondrial outer-membrane protein FUNDC1 mediates hypoxia-induced mitophagy in mammalian cells. Nat Cell Biol.

[149] Chen Z, Liu L, Cheng Q, Li Y, Wu H, Zhang W, Wang Y, Sehgal SA, Siraj S, Wang X, Wang J, Zhu Y, Chen Q (2017). Mitochondrial E3 ligase MARCH5 regulates FUNDC1 to fine-tune hypoxic mitophagy. EMBO Rep.

[150] Chen G, Han Z, Feng D, Chen Y, Chen L, Wu H, Huang L, Zhou C, Cai X, Fu C, Duan L, Wang X, Liu L, Liu X, Shen Y, Zhu Y, Chen Q (2014). A regulatory signaling loop comprising the PGAM5 phosphatase and CK2 controls receptor-mediated mitophagy. Mol Cell.

[151] Chen M, Chen Z, Wang Y, Tan Z, Zhu C, Li Y, Han Z, Chen L, Gao R, Liu L, Chen Q (2016). Mitophagy receptor FUNDC1 regulates mitochondrial dynamics and mitophagy. Autophagy.

[152] Wu S, Lu Q, Wang Q, Ding Y, Ma Z, Mao X, Huang K, Xie Z, Zou M-H (2017). Binding of FUN14 domain containing 1 with inositol 1,4,5-trisphosphate receptor in mitochondria-associated endoplasmic reticulum membranes maintains mitochondrial dynamics and function in hearts in vivo. Circulation.

[153] Wu H, Wang Y, Li W, Chen H, Du L, Liu D, Wang X, Xu T, Liu L, Chen Q (2019). Deficiency of mitophagy receptor FUNDC1 impairs mitochondrial quality and aggravates dietary-induced obesity and metabolic syndrome. Autophagy.

[154] Wang C, Dai X, Wu S, Xu W, Song P, Huang K (2021). FUNDC1-dependent mitochondria-associated endoplasmic reticulum membranes are involved in angiogenesis and neoangiogenesis. Nat Commun.

[155] Wu L, Zhang D, Zhou L, Pei Y, Zhuang Y, Cui W, Chen J (2019). FUN14 domain-containing 1 promotes breast cancer proliferation and migration by activating calcium-NFATC1-BMI1 axis. Ebiomedicine..

[156] Springer MZ, Poole LP, Drake LE, Bock-Hughes A, Boland ML, Smith AG, Hart J, Chourasia AH, Liu I, Bozek G, Macleod KF (2021). BNIP3-dependent mitophagy promotes cytosolic localization of LC3B and metabolic homeostasis in the liver. Autophagy.

[157] Vande Velde C, Cizeau J, Dubik D, Alimonti J, Brown T, Israels S, Hakem R, Greenberg AH (2000). BNIP3 and genetic control of necrosis-like cell death through the mitochondrial permeability transition pore. Mol Cell Biol.

[158] Burton TR, Gibson SB (2009). The role of Bcl-2 family member BNIP3 in cell death and disease: NIPping at the heels of cell death. Cell Death Differ.

[159] Metukuri MR, Beer-Stolz D, Namas RA, Dhupar R, Torres A, Loughran PA, Jefferso BS, Tsung A, Billiar TR, Vodovotz Y, Zamora R (2009). Expression and subcellular localization of BNIP3 in hypoxic hepatocytes and liver stress. Am J Physiol Gastrointest Liver Physiol.

[160] Graham RM, Thompson JW, Wei J, Bishopric NH, Webster KA (2007). Regulation of Bnip3 death pathways by calcium, phosphorylation, and hypoxia–reoxygenation. Antioxid Redox Signal.

[161] Bruick RK (2000). Expression of the gene encoding the proapoptotic Nip3 protein is induced by hypoxia. Proc Natl Acad Sci USA.

[162] Chourasia AH, Tracy K, Frankenberger C, Boland ML, Sharifi MN, Drake LE, Sachleben JR, Asara JM, Locasale JW, Karczmar GS, Macleod KF (2015). Mitophagy defects arising from BNip3 loss promote mammary tumor progression to metastasis. EMBO Rep.

[163] Jurasz P, Yurkova N, Kirshenbaum L, Stewart JD (2011). VEGF masks BNIP3-mediated apoptosis of hypoxic endothelial cells. Angiogenesis.

[164] Herkenne S, Ek O, Zamberlan M (2020). Developmental and tumor angiogenesis requires the mitochondria-shaping protein Opa1. Cell Metab.

[165] Lugus JJ, Ngoh GA, Bachschmid MM, Walsh K (2011). Mitofusins are required for angiogenic function and modulate different signaling pathways in cultured endothelial cells. J Mol Cell Cardiol.

[166] Gӧbel J, Engelhardt E, Pelzer P, Sakthivelu V, Jahn HM, Jevtic M, Folz-Donahue K, Kukat C, Schauss A, Frese CK, Giavalisco P, Ghanem A, Conzelmann K-K, Motori E, Bergami M (2020). Mitochondria-endoplasmic reticulum contacts in reactive astrocytes promote vascular remodeling. Cell Metab.

[167] Wang LT, He PC, Li AQ, Cao KX, Yan JW, Guo S, Jiang L, Yao L, Dai XY, Feng D, Xu YM, Tan N (2021). Caffeine promotes angiogenesis through modulating endothelial mitochondrial dynamics. Acta Pharmacol Sin.

[168] Shen T, Wang N, Yu X, Shi J, Li Q, Zhang C, Fu L, Wang S, Xing Y, Zheng X, Yu L, Zhu D (2015). The critical role of dynamin-related protein 1 in hypoxia-induced pulmonary vascular angiogenesis. J Cell Biochem.

[169] Lin J-R, Shen W-L, Yan C, Gao PJ (2015). Downregulation of dynamin-related protein 1 contributes to impaired autophagic flux and angiogenic function in senescent endothelial cells. Arterioscler Thromb Vasc Biol.

[170] Kim Y-M, Youn S-W, Sudhahar V, Das A, Chandhri R, Grajal HC, Kweon J, Leanhart S, He L, Toth PT, Kitajewski J, Rehman J, Yoon Y, Cho J, Fukai T, Ushio-Fukai M (2018). Redox regulation of mitochondrial fission protein Drp1 by protein disulfide isomerase limits endothelial senescence. Cell Rep.

[171] Zhou H, Shi C, Hu S, Zhu H, Ren J, Chen Y (2018). BI1 is associated with microvascular protection in cardiac ischemia reperfusion injury via repressing Syk–Nox2–Drp1-mitochondrial fission pathways. Angiogenesis.

[172] Wang H-H, Wu Y-J, Tseng Y-M, Su C-H, Hsieh C-L, Yeh H-I (2019). Mitochondrial fission protein 1 up-regulation ameliorates senescence-related endothelial dysfunction of human endothelial progenitor cells. Angiogenesis.

[173] Tatsuta T, Langer T (2017). Prohibitins. Curr Biol.

[174] Artal-Sanz M, Tavernarakis N (2009). Prohibitin and mitochondrial biology. Trends Endocrinol Metab.

[175] Salameh A, Daquinag AC, Staquicini DI, An Z, Hajjar KA, Pasqualini R, Arap W, Kolonin MG (2016). Prohibitin/annexin 2 interaction regulates fatty acid transport in adipose tissue. JCI Insight.

[176] Kolonin MG, Saha PK, Chan L, Pasqualini R, Arap W (2004). Reversal of obesity by targeted ablation of adipose tissue. Nat Med.

[177] Theiss AL, Idell RD, Srinivasan S, Klapproth J-M, Jones DP, Merlin D, Sitaraman SV (2007). Prohibitin protects against oxidative stress in intestinal epithelial cells. FASEB J.

[178] Theiss AL, Sitaraman SV (2011). The role and therapeutic potential of prohibitin in disease. Biochimi Biophys Acta Mol Cell Res.

[179] Xu Z, Wu J, Zha X (2011). Up-regulation of prohibitin 1 is involved in the proliferation and migration of liver cancer cells. Sci China Life Sci.

[180] Schleicher M, Shepherd BR, Suarez Y, Fernandez-Hernando C, Yu J, Pan Y, Acevedo LM, Shadel GS, Sessa WC (2008). Prohibitin-1 maintains the angiogenic capacity of endothelial cells by regulating mitochondrial function and senescence. J Cell Biol.

[181] Zhang M, Weng H, Zheng J (2019). NAD+ repletion inhibits the endothelial-to-mesenchymal transition induced by TGF-β in endothelial cells through improving mitochondrial unfolded protein response. Int J Biochem Cell Biol.

[182] Zhang H, Ryu D, Wu Y, Gariani K, Wang X, Luan P, D'Amico D, Ropelle ER, Lutolf MP, Aebersold R, Schoonjans K, Menzies KJ, Auwerx J (2016). NAD+ repletion improves mitochondrial and stem cell function and enhances life span in mice. Science.

[183] Mattiasson G, Sullivan PG (2006). The emerging functions of UCP2 in health, disease, and therapeutics. Antioxid Redox Signal.

[184] Vozza A, Parisi G, De Leonardis F, Lasorsa FM, Castegna A, Amorese D, Marmo R, Calcagnile VM, Palmieri L, Ricquier D, Paradies E, Scarcia P, Palmieri F, Bouillaud F, Fiermonte G (2014). UCP2 transports C4 metabolites out of mitochondria, regulating glucose and glutamine oxidation. Proc Natl Acad Sci USA.

[185] Diano S, Horvath TL (2012). Mitochondrial uncoupling protein 2 (UCP2) in glucose and lipid metabolism. Trends Mol Med.

[186] Xu M-J, Song P, Shirwany N, Liang B, Xing J, Viollet B, Wang X, Zhu Y, Zou M-H (2011). Impaired expression of uncoupling protein 2 causes defective postischemic angiogenesis in mice deficient in AMP-activated protein kinase α subunits. Arterioscler Thromb Vasc Biol.

[187] De Miguel C, Hamrick WC, Sedaka R, Jagarlamudi S, Asico LD, Jose PA, Cuevas S (2019). Uncoupling protein 2 increases blood pressure in DJ-1 knockout mice. J Am Heart Assoc.

[188] Shimasaki Y, Pan N, Messina LM, Li C, Chen K, Liu L, Cooper MP, Vita JA, Keaney JF (2013). Uncoupling protein 2 impacts endothelial phenotype via p53-mediated control of mitochondrial dynamics. Circ Res.

[189] Han X, Kong J, Hartnett ME, Wang H (2019). Enhancing retinal endothelial glycolysis by inhibiting UCP2 promotes physiologic retinal vascular development in a model of retinopathy of prematurity. Invest Ophthalmol Vis Sci.

[190] Pecqueur C, Bui T, Gelly C, Hauchard J, Barbot C, Bouillaud F, Ricquier D, Miroux B, Thompson CB (2008). Uncoupling protein-2 controls proliferation by promoting fatty acid oxidation and limiting glycolysis-derived pyruvate utilization. FASEB J.

[191] Samudio I, Fiegl M, McQueen T, Clise-Dwyer K, Andreeff M (2008). The warburg effect in leukemia-stroma cocultures is mediated by mitochondrial uncoupling associated with uncoupling protein 2 activation. Cancer Res.

[192] Zhang J, Khvorostov I, Hong JS, Oktay Y, Vergnes L, Nuebel E, Wahjudi PN, Setoguchi K, Wang G, Do A, Jung H-J, McCaffery JM, Kurland IJ, Reue K, Lee W-NP, Koehler CM, Teitell MA (2011). UCP2 regulates energy metabolism and differentiation potential of human pluripotent stem cells. EMBO J.

[193] Aguilar E, Esteves P, Sancerni T, Lenoir V, Aparicio T, Bouillaud F, Dentin R, Prip-Buus C, Ricquier D, Pecqueur C, Guilmeau S, Alves-Guerra M-C (2019). UCP2 deficiency increases colon tumorigenesis by promoting lipid synthesis and depleting NADPH for antioxidant defenses. Cell Rep.

[194] van de Ven RAH, Santos D, Haigis MC (2017). Mitochondrial sirtuins and molecular mechanisms of aging. Trends Mol Med.

[195] Yu W, Dittenhafer-Reed KE, Denu JM (2012). SIRT3 protein deacetylates isocitrate dehydrogenase 2 (IDH2) and regulates mitochondrial redox status. J Biol Chem.

[196] Ahn B-H, Kim H-S, Song S, Lee IH, Liu J, Vassilopoulos A, Deng C-X, Finkel T (2008). A role for the mitochondrial deacetylase Sirt3 in regulating energy homeostasis. Proc Natl Acad Sci USA.

[197] Shimazu T, Hirschey MD, Hua L, Dittenhafer-Reed KE, Schwer B, Lombard DB, Li Y, Bunkenborg J, Alt FW, Denu JM, Jacobson MP, Verdin E (2010). SIRT3 deacetylates mitochondrial 3-hydroxy-3-methylglutaryl CoA synthase 2 and regulates ketone body production. Cell Metab.

[198] Hallows WC, Yu W, Smith BC, Devries MK, Ellinger JJ, Someya S, Shortreed MR, Prolla T, Markley JL, Smith LM, Zhao S, Guan K-L, Denu JM (2011). Sirt3 promotes the urea cycle and fatty acid oxidation during dietary restriction. Mol Cell.

[199] Hirschey MD, Shimazu T, Goetzman E (2010). SIRT3 regulates mitochondrial fatty-acid oxidation by reversible enzyme deacetylation. Nature.

[200] Yang W, Nagasawa K, Münch C, Xu Y, Satterstrom K, Jeong S, Hayes SD, Jedrychowski MP, Vyas FS, Zaganjor E, Guarani V, Ringel AE, Gygi SP, Harper JW, Haigis MC (2016). Mitochondrial sirtuin network reveals dynamic SIRT3-dependent deacetylation in response to membrane depolarization. Cell.

[201] Dittenhafer-Reed Kristin E, Richards Alicia L, Fan J, Fan J, Smallegan MJ, Siahpirani AF, Kemmerer ZA, Prolla TA, Roy S, Coon JJ, Denu JM (2015). SIRT3 mediates multi-tissue coupling for metabolic fuel switching. Cell Metab.

[202] Rardin Matthew J, Newman John C, Held Jason M, Cusack MP, Sorensen DJ, Li B, Schilling B, Mooney SD, Kahn CR, Verdin E, Gibson BW (2013). Label-free quantitative proteomics of the lysine acetylome in mitochondria identifies substrates of SIRT3 in metabolic pathways. Proc Natl Acad Sci USA.

[203] Finley Lydia WS, Carracedo A, Lee J, Souza A, Egia A, Zhang J, Teruya-Feldstein J, Moreira PI, Cardoso SM, Clish CB, Pandolfi PP, Haigis MC (2011). SIRT3 opposes reprogramming of cancer cell metabolism through HIF1α destabilization. Cancer Cell.

[204] He J, Liu X, Su C, Wu F, Sun J, Zhang J, Yang X, Zhang C, Zhou Z, Zhang X, Lin X, Tao J (2019). Inhibition of mitochondrial oxidative damage improves reendothelialization capacity of endothelial progenitor cells via SIRT3 (Sirtuin 3)-enhanced SOD2 (superoxide dismutase 2) deacetylation in hypertension. Arterioscler Thromb Vasc Biol.

[205] Gao J, Wei T, Huang CL, Sun M, Shen W (2020). Sirtuin 3 governs autophagy-dependent glycolysis during angiotensin II-induced endothelial-to-mesenchymal transition. FASEB J.

[206] Mao X-B, You Z-P, Wu C, Huang J (2017). Potential suppression of the high glucose and insulin-induced retinal neovascularization by Sirtuin 3 in the human retinal endothelial cells. Biochem Biophys Res Commun.

[207] Zhang C, Li N, Suo M, Zhang C, Liu J, Liu L, Qi Y, Zheng X, Xie L, Hu Y, Bu P (2021). Sirtuin 3 deficiency aggravates angiotensin II-induced hypertensive cardiac injury by the impairment of lymphangiogenesis. J Cell Mol Med.

[208] Wei T, Huang G, Gao J, Huang C, Sun M, Wu J, Bu J, Shen W (2017). Sirtuin 3 deficiency accelerates hypertensive cardiac remodeling by impairing angiogenesis. J Am Heart Assoc.

[209] He X, Zeng H, Chen ST, Roman RJ, Aschner JL, Didion S, Chen JX (2017). Endothelial specific SIRT3 deletion impairs glycolysis and angiogenesis and causes diastolic dysfunction. J Mol Cell Cardiol..

[210] Kim S, Piao S, Lee I, Nagar H, Choi SJ, Shin N, Kim DW, Shong M, Jeon BH, Kim CS (2020). CR6 interacting factor 1 deficiency induces premature senescence via SIRT3 inhibition in endothelial cells. Free Radic Biol Med.

[211] Dikalova AE, Pandey A, Xiao L, Arslanbaeva L, Sidorova T, Lopez MG, Billings FT, Verdin E, Auwerx J, Harrison DG, Dikalov SI (2020). Mitochondrial deacetylase Sirt3 reduces vascular dysfunction and hypertension while Sirt3 depletion in essential hypertension is linked to vascular inflammation and oxidative stress. Circ Res.

[212] Mathias RA, Greco TM, Oberstein A, Budayeva HG, Chakrabarti R, Rowland EA, Kang Y, Shenk T, Cristea IM (2014). Sirtuin 4 Is a lipoamidase regulating pyruvate dehydrogenase complex activity. Cell.

[213] Laurent G, German NJ, Saha AK (2013). SIRT4 coordinates the balance between lipid synthesis and catabolism by repressing malonyl CoA decarboxylase. Mol Cell.

[214] Li K, Hüsing A, Fortner RT (2015). An epidemiologic risk prediction model for ovarian cancer in Europe: the EPIC study. Br J Cancer.

[215] Tomaselli D, Steegborn C, Mai A, Rotili D (2020). Sirt4: a multifaceted enzyme at the crossroads of mitochondrial metabolism and cancer. Front Oncol..

[216] Tao Y, Huang C, Huang Y, Hong L, Wang H, Zhou Z, Qiu Y (2015). SIRT4 suppresses inflammatory responses in human umbilical vein endothelial cells. Cardiovasc Toxicol.

[217] Sadhukhan S, Liu X, Ryu D, Nelson OD, Stupinski J, Li Z, Chen W, Zhang S, Weiss RS, Locasale JW, Auwerx J, Lin H (2016). Metabolomics-assisted proteomics identifies succinylation and SIRT5 as important regulators of cardiac function. Proc Natl Acad Sci USA.

[218] Nakagawa T, Lomb DJ, Haigis MC, Guarente L (2009). SIRT5 deacetylates carbamoyl phosphate synthetase 1 and regulates the urea cycle. Cell.

[219] Nishida Y, Rardin MJ, Carrico C, He W, Sahu AK, Gut P, Najjar R, Fitch M, Hellerstein M, Gibson BR, Verdin E (2015). SIRT5 regulates both cytosolic and mitochondrial protein malonylation with gycolysis as a major target. Mol Cell.

[220] Rardin MJ, He W, Nishida Y, Newman JC, Carrico C, Danielson SR, Guo A, Gut P, Sahu AK, Li B, Uppala R, Fitch M, Riiff T, Zhu L, Zhou J, Mulhern D, Stevens RD, Ilkayeva OR, Newgard CB, Jacobson MP, Hellerstein M, Goetzman ES, Gibson BW, Verdin E (2013). SIRT5 regulates the mitochondrial lysine succinylome and metabolic networks. Cell Metab.

[221] Yu BB, Zhi H, Zhang XY, Liang JW, He J, Su C, Xia WH, Zhang GX, Tao J (2019). Mitochondrial dysfunction-mediated decline in angiogenic capacity of endothelial progenitor cells is associated with capillary rarefaction in patients with hypertension via downregulation of CXCR4/JAK2/SIRT5 signaling. EBioMedicine.

[222] Shang B, Xu T, Hu N, Mao Y, Du X (2021). Circ-Klhl8 overexpression increased the therapeutic effect of EPCs in diabetic wound healing via the miR-212-3p/SIRT5 axis. J Diabetes Complicat.

[223] Diaz-Cañestro C, Merlini M, Bonetti NR, Liberale L, Wüst P, Briand-Schumacher S, Klohs J, Costantino S, Miranda M, Schoedon-Geiser G, Kullak-Ublick GA, Akhmedov A, Paneni F, Beer JH, Lüscher TF, Camici GG (2018). Sirtuin 5 as a novel target to blunt blood brain barrier damage induced by cerebral ischemia/reperfusion injury. Int J Cardiol..

[224] Frömel T, Naeem Z, Pirzeh L, Fleming I (2022). Cytochrome P450-derived fatty acid epoxides and diols in angiogenesis and stem cell biology. Pharmacol Ther.

[225] Omura T, Gotoh O (2017). Evolutionary origin of mitochondrial cytochrome P450. J Biochem.

[226] Cui P, Luo Z, Zhang H, Su Y, Li A, Li H, Zhang J, Yang Z, Xiu R (2006). Effect and mechanism of melatonin's action on the proliferation of human umbilical vein endothelial cells. J Pineal Res.

[227] Jobe SO, Ramadoss J, Koch JM, Jiang Y, Zheng J, Magness RR (2010). Estradiol-17β and its cytochrome P450- and catechol-O-methyltransferase–derived metabolites stimulate proliferation in uterine artery endothelial cells. Hypertension.

[228] Armulik A, Genové G, Betsholtz C (2011). Pericytes: developmental, physiological, and pathological perspectives, problems, and promises. Dev Cell.

[229] Tang Y, Scheef EA, Wang S, Sorenson CM, Marcus CB, Jefcoate CR, Sheibani N (2009). CYP1B1 expression promotes the proangiogenic phenotype of endothelium through decreased intracellular oxidative stress and thrombospondin-2 expression. Blood.

[230] Falero-Perez J, Sorenson CM, Sheibani N (2019). Cyp1b1-deficient retinal astrocytes are more proliferative and migratory and are protected from oxidative stress and inflammation. Am J Physiol Cell Physiol.

[231] Palenski TL, Sorenson CM, Jefcoate CR, Sheibani N (2013). Lack of Cyp1b1 promotes the proliferative and migratory phenotype of perivascular supporting cells. Lab Invest.

[232] Falero-Perez J, Larsen MC, Teixeira LBC, Zhang HF, Lindner V, Sorenson CM, Jefcoate CR, Sheibani N (2019). Targeted deletion of Cyp1b1 in pericytes results in attenuation of retinal neovascularization and trabecular meshwork dysgenesis. Trends Dev Biol.

[233] Palenski TL, Gurel Z, Sorenson CM, Hankenson KD, Sheibani N (2013). Cyp1B1 expression promotes angiogenesis by suppressing NF-κB activity. Am J Physiol Cell Physiol.

[234] Tang Y, Scheef EA, Gurel Z, Sorenson CM, Jefcoate CR, Sheibani N (2009). CYP1B1 and endothelial nitric oxide synthase combine to sustain proangiogenic functions of endothelial cells under hyperoxic stress. Am J Physiol Cell Physiol.

[235] Falero-Perez J, Song Y-S, Zhao Y, Teixeira L, Sorenson CM, Sheibani N (2018). Cyp1b1 expression impacts the angiogenic and inflammatory properties of liver sinusoidal endothelial cells. PLoS One.

[236] Merrill RA, Strack S (2014). Mitochondria: A kinase anchoring protein 1, a signaling platform for mitochondrial form and function. Int J Biochem Cell Biol.

[237] Livigni A, Scorziello A, Agnese S, Adornetto A, Carlucci A, Garbi C, Castaldo I, Annunziato L, Avvedimento EV, Feliciello A (2005). Mitochondrial AKAP121 links cAMP and src signaling to oxidative metabolism. Mol Biol Cell.

[238] Perrino C, Feliciello A, Schiattarella GG, Esposito G, Guerriero R, Zaccaro L, Gatto AD, Saviano M, Garbi C, Carangi R, Lorenzo ED, Donato G, Indolfi C, Avvedimento VE, Chiariello M (2010). AKAP121 downregulation impairs protective cAMP signals, promotes mitochondrial dysfunction, and increases oxidative stress. Cardiovasc Res.

[239] Ji L, Zhao Y, He L, Zhao J, Gao T, Liu F, Qi B, Kang F, Wang G, Zhao Y, Guo H, He Y, Li F, Huang Q, Xing J (2021). AKAP1 deficiency attenuates diet-induced obesity and insulin resistance by promoting fatty acid oxidation and thermogenesis in brown adipocytes. Adv Sci..

[240] Aggarwal S, Gabrovsek L, Langeberg LK, Golkowski M, Ong S-E, Smith FD, Scott JD (2019). Depletion of dAKAP1–protein kinase A signaling islands from the outer mitochondrial membrane alters breast cancer cell metabolism and motility. J Biol Chem.

[241] Rinaldi L, Sepe M, Delle Donne R, Conte K, Arcella A, Borzacchiello D, Amente S, De Vita F, Porpora M, Garbi C, Oliva MA, Procaccini C, Faicchia D, Matarese G, Marino FZ, Rocco G, Pignatiello S, Franco R, Insabato L, Majello B, Feliciello A (2017). Mitochondrial AKAP1 supports mTOR pathway and tumor growth. Cell Death Dis.

[242] Meyer RD, Srinivasan S, Singh AJ, Mahoney JE, Gharahassanlou KR, Rahimi N (2011). PEST motif serine and tyrosine phosphorylation controls vascular endothelial growth factor receptor 2 stability and downregulation. Mol Cell Biol.

[243] Schiattarella GG, Cattaneo F, Carrizzo A (2018). Akap1 regulates vascular function and endothelial cells behavior. Hypertension.

[244] Sun F, Cheng Y, Chen C (2015). Regulation of heme biosynthesis and transport in metazoa. Sci China Life Sci.

[245] Chen W, Dailey HA, Paw BH (2010). Ferrochelatase forms an oligomeric complex with mitoferrin-1 and Abcb10 for erythroid heme biosynthesis. Blood.

[246] Patel D, Alhawaj R, Kelly MR, Accarino JJO, Lakhkar A, Gupte SA, Sun D, Wolin MS (2016). Potential role of mitochondrial superoxide decreasing ferrochelatase and heme in coronary artery soluble guanylate cyclase depletion by angiotensin II. Am J Physiol Heart Circ Physiol.

[247] Basavarajappa HD, Sulaiman RS, Qi X, Shetty T, Sheik Pran Babu S, Sishtla KL, Lee B, Quigley J, Alkhairy S, Briggs CM, Gupta K, Tang B, Shadmand M, Grant MB, Boulton ME, Seo SY, Corson TW (2017). Ferrochelatase is a therapeutic target for ocular neovascularization. EMBO Mol Med.

[248] Pran Babu SPS, White D, Corson TW (2020). Ferrochelatase regulates retinal neovascularization. FASEB J.

[249] Shetty T, Sishtla K, Park B, Repass MJ, Corson TW (2020). Heme synthesis inhibition blocks angiogenesis via mitochondrial dysfunction. iScience..

[250] Sishtla K, Lambert-Cheatham N, Lee B, Han DH, Park J, Sardar Pasha SPB, Lee S, Kwon S, Muniyandi A, Park B, Odell N, Waller S, IlY P, Lee SJ, Seo S-Y, Corson TW (2022). Small-molecule inhibitors of ferrochelatase are antiangiogenic agent. Cell Chem Biol.

[251] Wang L, Astone M, Alam SK, Zhu Z, Pei W, Frank DA, Burgess SM, Hoeppner LH (2021). Suppressing STAT3 activity protects the endothelial barrier from VEGF-mediated vascular permeability. Dis Model Mech.

[252] Hoffmann CJ, Harms U, Rex A, Szulzewsky F, Wolf SA, Grittner U, Lättig-Tünnemann G, Sendtner M, Kettenmann H, Dirnagl U, Endres M, Harms C (2015). Vascular signal transducer and activator of transcription-3 promotes angiogenesis and neuroplasticity long-term after stroke. Circulation.

[253] Zouein FA, Booz GW, Altara R (2019). STAT3 and endothelial cell—cardiomyocyte dialog in cardiac remodeling. Front Cardiovasc Med.

[254] Comità S, Femmino S, Thairi C, Alloatti G, Boengler K, Pagliaro P, Penna C (2021). Regulation of STAT3 and its role in cardioprotection by conditioning: focus on non-genomic roles targeting mitochondrial function. Basic Res Cardiol.

[255] Wegrzyn J, Potla R, Chwae YJ, Sepuri NB, Zhang Q, Koeck T, Derecka M, Szczepanek K, Szelag M, Gornicka A, Moh A, Moghaddas S, Chen Q, Bobbili S, Cichy J, Dulak J, Baker DP, Wolfman A, Stuehr D, Hassan MO, Fu XY, Avadhani N, Drake JI, Fawcett P, Lesnefsky EJ, Larner AC (2009). Function of mitochondrial Stat3 in cellular respiration. Science.

[256] Tammineni P, Anugula C, Mohammed F, Anjaneyulu M, Larner AC, Sepuri NBV (2013). The import of the transcription factor STAT3 into mitochondria depends on GRIM-19, a component of the electron transport chain. J Biol Chem.

[257] Mohammed F, Gorla M, Bisoyi V, Tammineni P, Sepuri NBV (2020). Rotenone-induced reactive oxygen species signal the recruitment of STAT3 to mitochondria. FEBS Lett.

[258] Hu C, Wu Z, Huang Z, Hao X, Wang S, Deng J, Yin Y, Tan C (2021). Nox2 impairs VEGF-A-induced angiogenesis in placenta via mitochondrial ROS-STAT3 pathway. Redox Bio.

[259] Murga M, Fernandez-Capetillo O, Tosato G (2005). Neuropilin-1 regulates attachment in human endothelial cells independently of vascular endothelial growth factor receptor-2. Blood.

[260] Roth L, Prahst C, Ruckdeschel T, Savant S, Weström S, Fantin A, Riedel M, Héroult M, Ruhrberg C, Augustin HG (2016). Neuropilin-1 mediates vascular permeability independently of vascular endothelial growth factor receptor-2 activation. Sci Signal..

[261] Fantin A, Vieira JM, Plein A, Denti L, Fruttiger M, Pollard JW, Ruhrberg C (2013). NRP1 acts cell autonomously in endothelium to promote tip cell function during sprouting angiogenesis. Blood.

[262] Fantin A, Lampropoulou A, Gestri G, Raimondi C, Senatore V, Zachary I, Ruhrberg C (2015). NRP1 regulates CDC42 activation to promote filopodia formation in endothelial tip cells. Cell Rep.

[263] Bae D, Lu S, Taglienti CA, Mercurio AM (2008). Metabolic stress induces the lysosomal degradation of neuropilin-1 but not neuropilin-2. J Biol Chem.

[264] Issitt T, Bosseboeuf E, De Winter N, Dufton N, Gestri G, Senatore V, Chikh A, Randi AM, Raimondi C (2019). Neuropilin-1 controls endothelial homeostasis by regulating mitochondrial function and iron-dependent oxidative stress. iScience..

[265] Foster MN, Coetzee WA (2016). KATP channels in the cardiovascular system. Physiol Rev.

[266] Blanco-Rivero J, Gamallo C, Aras-López R, Cobeño L, Cogolludo A, Pérez-Vizcaino F, Ferrer M, Balfagon G (2008). Decreased expression of aortic KIR6.1 and SUR2B in hypertension does not correlate with changes in the functional role of KATP channels. Eur J Pharmacol..

[267] Fedele F, Mancone M, Chilian WM, Severino P, Canali E, Logan S, De Marchis ML, Volterrani M, Palmirotta R, Guadagni F (2013). Role of genetic polymorphisms of ion channels in the pathophysiology of coronary microvascular dysfunction and ischemic heart disease. Basic Res Cardiol.

[268] Bukar U, Anastasia P, Vasileios K, Andreas P, Stavros T (2015). ATP-sensitive potassium channel activation induces angiogenesis in vitro and in vivo. J Pharmacol Exp Ther.

[269] Wu Y, He MY, Ye JK, Ma SY, Huang W, Wei YY, Kong H, Wang H, Zeng XN, Xie WP (2017). Activation of ATP-sensitive potassium channels facilitates the function of human endothelial colony-forming cells via Ca2+/Akt/eNOS pathway. J Cell Mol Med.

[270] Aziz Q, Li Y, Anderson N, Ojake L, Tsisanova E, Tinker A (2017). Molecular and functional characterization of the endothelial ATP-sensitive potassium channel. J Biol Chem.

[271] Li Y, Aziz Q, Anderson N, Ojake L, Tinker A (2020). Endothelial ATP-sensitive potassium channel protects against the development of hypertension and atherosclerosis. Hypertension.

[272] Forini F, Lionetti V, Ardehali H, Pucci A, Cecchetti F, Ghanefar M, Nicolini G, Ichikawa Y, Nannipieri M, Recchia FA, Iervasi G (2011). Early long-term L-T3 replacement rescues mitochondria and prevents ischemic cardiac remodelling in rats. J CellL Mol Med.

[273] Todorova D, Simoncini S, Lacroix R, Sabatier F, Dignat-George F (2017). Extracellular vesicles in angiogenesis. Circ Res.

[274] Moeinabadi-Bidgoli K, Rezaee M, Hossein-Khannazer N, Babajani A, Aghdaei HA, Arki MK, Afaghi S, Niknejad H, Vosough M (2023). Exosomes for angiogenesis induction in ischemic disorders. J Cell Mol Med.

[275] Chance TC, Herzig MC, Christy BA, Delavan C, Rathbone CR, Cap AP, Bynum JA (2020). Human mesenchymal stromal cell source and culture conditions influence extracellular vesicle angiogenic and metabolic effects on human endothelial cells in vitro. J Trauma..

[276] Zhang Y, Bai X, Shen K, Luo L, Zhao M, Xu C, Jia Y, Xiao D, Li Y, Gao X, Tian C, Wang Y, Hu D (2022). Exosomes derived from adipose mesenchymal stem cells promote diabetic chronic wound healing through SIRT3/SOD2. Cells..

[277] Ma X, Wang J, Li J, Ma C, Chen S, Lei W, Yang Y, Liu S, Bihl J, Chen C (2018). Loading MiR-210 in endothelial progenitor cells derived exosomes boosts their beneficial effects on hypoxia/reoxygeneation-injured human endothelial cells via protecting mitochondrial function. Cell Physiol Biochem.

[278] Hayakawa K, Chan SJ, Mandeville ET, Park JH, Bruzzese M, Montaner J, Arai K, Rosell A, Lo EH (2018). Protective effects of endothelial progenitor cell-derived extracellular mitochondria in brain endothelium. Stem Cells.

[279] D'Souza A, Burch A, Dave KM, Sreeram A, Reynolds MJ, Dobbins DX, Kamte YS, Zhao W, Sabatelle C, Joy GM, Soman V, Chandran UR, Shiva SS, Quillinan N, Herson PS, Manickam DS (2021). Microvesicles transfer mitochondria and increase mitochondrial function in brain endothelial cells. J Control Release.

[280] Birkeland KI, Jørgensen ME, Carstensen B, Persson F, Gulseth HL, Thuresson M, Fenici P, Nathanson D, Nyström T, Eriksson JW, Bodegård J, Norhammar A (2017). Cardiovascular mortality and morbidity in patients with type 2 diabetes following initiation of sodium-glucose co-transporter-2 inhibitors versus other glucose-lowering drugs (CVD-REAL Nordic): a multinational observational analysis. Lancet Diabetes Endo.

[281] Cowie MR, Fisher M (2020). SGLT2 inhibitors: mechanisms of cardiovascular benefit beyond glycaemic control. Nat Rev Cardiol.

[282] Zhou H, Wang S, Zhu P, Hu S, Chen Y, Ren J (2018). Empagliflozin rescues diabetic myocardial microvascular injury via AMPK-mediated inhibition of mitochondrial fission. Redox Biol.

[283] Mone P, Varzideh F, Jankauskas SS, Pansini A, Lombardi A, Frullone S, Santulli G (2022). SGLT2 inhibition via empagliflozin improves endothelial function and reduces mitochondrial oxidative stress: insights from frail hypertensive and diabetic patients. Hypertension.

[284] Behnammanesh G, Durante ZE, Peyton KJ, Martinez-Lemus LA, Brown SM, Bender SB, Durante W (2019). Canagliflozin inhibits human endothelial cell proliferation and tube formation. Front Pharmacol.

